# Overexpression of Cytokinin Dehydrogenase Genes in Barley (*Hordeum vulgare* cv. Golden Promise) Fundamentally Affects Morphology and Fertility

**DOI:** 10.1371/journal.pone.0079029

**Published:** 2013-11-15

**Authors:** Katarína Mrízová, Eva Jiskrová, Šárka Vyroubalová, Ondřej Novák, Ludmila Ohnoutková, Hana Pospíšilová, Ivo Frébort, Wendy A. Harwood, Petr Galuszka

**Affiliations:** 1 Department of Molecular Biology, Centre of the Region Haná for Biotechnological and Agricultural Research, Palacký University, Olomouc, Czech Republic; 2 Laboratory of Growth Regulatory, Palacký University and Institute of Experimental Botany, Olomouc, Czech Republic; 3 Department of Crop Genetics, John Innes Centre, Norwich, United Kingdom; University of Nottingham, United Kingdom

## Abstract

Barley is one of the most important cereal crops grown worldwide. It has numerous applications, but its utility could potentially be extended by genetically manipulating its hormonal balances. To explore some of this potential we identified gene families of cytokinin dehydrogenases (CKX) and isopentenyl transferases, enzymes that respectively irreversibly degrade and synthesize cytokinin (CK) plant hormones, in the raw sequenced barley genome. We then examined their spatial and temporal expression patterns by immunostaining and qPCR. Two CKX-specific antibodies, anti-HvCKX1 and anti-HvCKX9, predominantly detect proteins in the aleurone layer of maturing grains and leaf vasculature, respectively. In addition, two selected *CKX* genes were used for stable, *Agrobacterium tumefaciens*-mediated transformation of the barley cultivar Golden Promise. The results show that constitutive overexpression of *CKX* causes morphological changes in barley plants and prevents their transition to flowering. In all independent transgenic lines roots proliferated more rapidly and root-to-shoot ratios were higher than in wild-type plants. Only one transgenic line, overexpressing *CKX* under the control of a promoter from a phosphate transporter gene, which is expressed more strongly in root tissue than in aerial parts, yielded progeny. Analysis of several T1-generation plants indicates that plants tend to compensate for effects of the transgene and restore CK homeostasis later during development. Depleted CK levels during early phases of development are restored by down-regulation of endogenous *CKX* genes and reinforced *de novo* biosynthesis of CKs.

## Introduction

Genetic engineering is a useful approach for creating crop plants with desired qualities. Since cereal species have immense agricultural importance, there have been many recent advances in transformation techniques for monocot plants [1] allowing rapid development of novel transgenic varieties. Barley (*Hordeum vulgare* L.) is a widely grown cereal with valuable traits, including high malting quality and nutritional value, which are exploited in brewing, distilling and the production of diverse human and animal foods. Genetic engineering has already been used in research focused on improving barley characteristics such as its malting quality and disease resistance [2]. Barley seeds have also been used as bioreactors for molecular farming (for a review see Dunwell [3]). Companies such as ORF Genetics (Iceland) and Maltagen Forschung GmbH (Germany) have started to produce pharmaceutical proteins (*inter alia* growth factors, cytokines, oral vaccines and food additives) by robust, endosperm-driven expression in transgenic barley lines [4].

Various transgenic manipulations designed to perturb hormonal balances of cereals could also be beneficial in agricultural industry, for example ectopic overexpression or silencing of genes encoding cytokinin dehydrogenase (CKX; EC 1.5.99.12), a key enzyme in the regulation of cytokinin (CK) homeostasis responsible for irreversible degradation of CKs [5]. This enzyme preferentially cleaves isoprenoid types of CKs to adenine and an aldehyde derived from the isoprenoid side chain. Appropriately balanced levels of CKs in plants are important for promoting cell division locally, ensuring correct organ differentiation and directing numerous physiological processes [6]. These balances are homeostatically maintained by complex regulation of differential basipetal and acropetal transport of isoprenoid CKs, degradation by CKX, reversible conjugation to inactive glucosides and direct biosynthesis *in situ* by the activity of isopentenyl transferases (IPTs). Plant genomes contain small gene families encoding *IPT* and *CKX* genes, that show substantial differences in spatial and temporal expression patterns.

Several typical morphological perturbations associated with CK over-accumulation or deficiency in plants have been described, including the following. Accumulation of CKs in inflorescence meristems, caused by the mutation in promoter region of the *OsCKX2* gene in the Habataki rice variety, reportedly leads to increases in numbers of reproductive organs and hence grains per panicle [7]. Transgenic tobacco and Arabidopsis plants constitutively expressing Arabidopsis *CKX* genes display phenotypic alterations including severely retarded shoot growth and significantly enhanced root growth [6], [8]. Shoot sink tissues of CK-deficient tobacco plants have reduced activities of vacuolar invertases and contents of both soluble sugars and ATP [9]. These findings clearly indicate that CKs play an important role in the maintenance of shoot sink strength. Furthermore, CK deficiency reduces the activity of vegetative and floral shoot apical meristems, leading to retarded shoot development. In contrast, it enhances root system development by delaying the entry of dividing cells into the elongation phase, thus increasing their accumulation in the meristem [10]. CK-deficient plants also initiate more lateral root primordia, which elongate more rapidly than those of wild-type plants [6].

Here, we examine whole gene families encoding two major groups of CK biosynthetic and deactivation enzymes, together with their distribution patterns in barley plants. Further, we show that reduction of CK levels during an early phase of development leads to similar morphological alterations in monocot plants to those previously described for model dicot plants. However, there are probably differences in their CK-regulating mechanisms as different CK forms are prevalent in barley tissues and their alteration via *CKX* overexpression has stronger negative effects on flowering than in tobacco or Arabidopsis.

## Materials and Methods

### Immunohistochemical detection of CKX

We amplified a 420-bp fragment of *HvCKX1* using 5′-cctcatccctggctcaacgtgctc-3′ and 5′-ttagttgaagatgtcttggcccggggag-3 primers, and a 735-bp fragment of *HvCKX9* using 5′-ctaaacaactggaggtcatcgttc-3′ and 5′-ttacagtaggtactgtaacgaggacaa-3′ primers. The respective amplicons were subcloned into the pCRT7/NT-TOPO vector and resulting constructs were used to prepare specific antigens in *E. coli* BL21 (DE3) Star (Invitrogen) that were purified from crude cell lysate on Ni-NTA Sepharose HP (GE-Healthcare). To prepare the antibodies, 3 mg of purified recombinant protein was mixed with Freud's complete adjuvant at a 1∶1 ratio and used to inject New Zealand white rabbits (two per experiment) intramuscularly five times (dosages: 1, 1, 0.5, 0.25 and 0.25 mg) during a period of 8 weeks. The rabbits were bled a week after the final injection and respective polyclonal CKX antisera were purified using an affinity matrix consisting of recombinant HvCKX1 or HvCKX9 fragments coupled to Sepharose 4B (Sigma) according to the supplier's recommendations.

Plant tissue (trimmed grains, leaf and root segments) were fixed in 4% paraformaldehyde and 0.2% glutaraldehyde in PBS for 16 hours, at 4°C under vacuum and then embedded in Paraplast (Sigma). Sections (15–25 µm) were cut using a microtome and mounted on positively charged slides. Tissue sections were deparaffinized in xylene, rehydrated in a graded ethanol series and rinsed with PBS. After overnight blocking in PBS containing 5% bovine powdered milk, the sections were incubated with the purified CKX antiserum (1∶1,000) for 2 hours and then an anti-rabbit secondary antibody conjugated with alkaline phosphatase (1∶1000; Sigma). CKX proteins were visualized with a Fast Red TR/Napthol AS-MX kit (Sigma). Control samples were prepared accordingly without adding the primary antibody.

The polyclonal antibodies were produced in accordance with current Czech legislation (Animal Protection and Welfare Act No. 246/1992 Coll. of the Government of the Czech Republic). The specific experiments were approved by the Ethics Committee of the Faculty of Sciences, Palacký University, Olomouc (permit number 02/2003) and the Committee for Animal Welfare of the Ministry of Agriculture of the Czech Republic (permit number 18 172/2003-30/300).

### Agrobacterium-mediated transformation of barley

The genomic sequences of *HvCKX9* (AF490591) and cDNA of *ZmCKX1* (NM_001112121) were amplified from previously prepared vectors [11], [12] by Phusion DNA-polymerase (New England Biolabs) using the following primers: *gHvCKX9* (5′-cggggtaccatgaggcaattactcctgcaa-3′ and 5′-tgctctagattacagtaggtactgtaacgaggac-3′); *ZmCKX1* (5′-acgcgtcgacatggcggtggtttattacctgctgct-3′ and 5′-ccgctcgagtcatcagttgaagatgtcctggc-3′). *HvCKX9* and *ZmCKX1* amplicons with KpnI/XbaI and SalI/XhoI overhangs, respectively, were subcloned into the pENTR1A vector and sequenced. The genes were inserted into the pBRACT214 vector downstream of the ubiquitin (*Ubi*) promoter (John Innes Center, Norwich, UK; www.bract.org) via Gateway recombination (Invitrogen). Phusion DNA-polymerase was also used to amplify the sequences of promoters of a barley phosphate transporter (PHT1-1; AF543197) [13] with 5′-gagctccgactacccccgcgata-3′ and 5′-ggtcgccggcgatctctcagc-3′, and barley root abundant factor (RAF; DQ102384) [14] with 5′-ggttaggagagtggttatagcccaagcc-3′ and 5′-tgttttatcttcctgtctcgcgct-3′ primers, from barley genomic DNA. Amplified promoter sequences were inserted into the pDrive vector (Qiagen), verified by sequencing then subcloned into the pCambia1281Z vector upstream of the β-glucuronidase (*GUS*) gene. The *ZmCKX1* gene and the nopaline synthase (NOS) terminator were amplified by Phusion DNA-polymerase from the pROM30 vector [12], sequenced and inserted between SalI and HindIII restriction sites into the pDrive vector downstream of the *PHT1-1* or *RAF* promoter. Whole cassettes containing the promoter, gene and terminator were re-cloned from the pDrive vector into the pENTR1 vector through KpnI and XhoI restriction sites then inserted into pBRACT209 (John Innes Center, Norwich) via Gateway recombination. The final vectors were electro-transformed into *Agrobacterium tumefaciens* strain AGL1 together with the helper vector pSoup.

Arabidopsis Col-0 plants were transformed by the flower-dip method [15]. The barley transformation procedure was modified from protocols described by Cho [16] and Bartlett [17]. All media were prepared according to Bartlett [17]. Plants of the spring barley, cultivar Golden Promise, were grown in an environmental chamber with the photoperiod of 15°C/16 hours/light and 12°C/8 hours/dark. The light source was a combination of mercury tungsten lamps and sodium lamps providing the intensity of 500 μmol m^−2^ s^−1^. The plants were cultivated in a 2∶1 mixture of soil and perlite (Perlit Ltd., Czech Republic) and fertilized every 14 days. Immature seeds were collected, washed in 70% ethanol, sterilized in 6% sodium hypochlorite for 25 minutes then washed in sterile water. Immature embryos, 1.5–2 mm in diameter, were exposed by scalpel and forceps then embryogenic axes were removed. The immature embryos were placed scutellum side down on a callus induction medium supplemented with 2.5 mg l^−1^ of dicamba, 5 mmol l^−1^ of CuSO_4_ and cultivated overnight in the dark at 26°C. *Agrobacterium* culture (OD_600_ = 0.6) bearing the respective vector was re-suspended in Murashige-Skoog (Sigma) medium supplemented with 200 μM acetosyringone. Each immature embryo was covered with a drop of the *Agrobacterium* suspension and then placed scutellum side down on callus induction medium supplemented with 2.5 mg l^−1^ of dicamba and 5 mmol l^−1^ of CuSO_4_. After 3 days of the cultivation at 26°C in the dark, the embryos were transferred scutellum side down on the callus induction medium supplemented with 2.5 mg l^−1^ dicamba, 5 mmol l^−1^ of CuSO_4_, 150 mg l^−1^ Timentin, and 50 mg l^−1^ hygromycin (Roche). The embryos were then cultivated in the dark at 26°C and transferred onto fresh medium every 2 weeks. After 6 weeks, the embryos were transferred onto a regeneration medium supplemented with 2.5 mg l^−1^ of 2,4-dichlorophenoxyacetic acid, 1 mg l^−1^ *N* ^6^-benzylaminopurine (BAP) and placed into an environmental chamber with a low light intensity (80 μmol m^−2^ s^−1^) with the photoperiod of 24°C/16 hours/light and 22°C/8 hours/dark. After additional 2 to 4 weeks, embryogenic calli were transferred onto a regeneration medium supplemented with 1 mg l^−1^ BAP and placed into a chamber with full light (160 μmol m^−2^ s^−1^). Well-developed shoots were rooted on the hormone-free callus induction medium. Plantlets with a developed root system were placed in soaked jiffy pellets (A/S Jiffy Products, Norway) and acclimatized using the same conditions as during the regeneration phase. After a week of acclimatization the plants were transferred to soil and grown in the environmental chamber, again providing 24°C/16 hour light and 22°C/8 hour dark cycles with low light intensity (80 μmol m^−2^ s^−1^) during the light phases.

### qPCR analysis

cDNA was obtained from the RNA using a *RevertAid* First Strand cDNA Synthesis Kit (Fermentas), then amplified by qPCR in 10 μl reaction mixtures containing TaqMan Gene Expression Master Mix or Power SYBR Green PCR Master Mix (Life Technologies), 300 nM of each primer and, when the TaqMan mixture was used, 250 nM specific 5′6-carboxyfluorescein (FAM) dye and 3′5 (6)-carboxytetramethylrhodamine (TAMRA) quencher. Reactions were run in a ViiA7 Real-Time PCR System using a default program (Applied Biosystems). Primers and Taqman probes were designed using Primer Express 3.0 software (Tables S1 and S2). Genes encoding β-actin and elongation factor 1 were selected as the most consistently expressed and used as references for samples from all tissues. Amplicons for every primer pair used were produced by standard Taq polymerase, cloned into the pDrive vector, and sequenced by a commercial sequencing service. For each pair of primers, plasmid DNA was subsequently used as a template to generate a calibration curve for determining the efficiency of PCR and subtracting number of transcripts per defined amount of isolated total RNA.

### CKX activity assay

To measure CKX activity, protein extracts were obtained by freezing plant samples in liquid nitrogen, powdering them with a mortar and pestle, extracting with 0.2 M Tris/HCl buffer (pH 8.0) containing 1 mM phenylmethylsulfonylfluoride and 0.3% Triton X-100, then removing cell debris by centrifugation at 21,000 g for 10 minutes. The samples' CKX activities were determined in triplicate assays by spectrophotometrically measuring products yielded by incubating 5–30 µl of protein extract at 37°C in 0.5 ml reaction mixtures containing 100 mM McIlvaine buffer (pH 6.0), 0.25 mM *N* ^6^-isopentenyladenine (iP) or *N* ^6^-isopentenyladenine-9-glucoside (IP9G) as substrate and 0.5 mM 2,3-dimethoxy-5-methyl-1,4-benzoquinone as an electron acceptor [18]. In addition, the samples' protein contents were estimated following Bradford [19] with bovine serum albumin as the standard.

### Western blot analysis

To detect transgenic protein, extracts from the plants concentrated on a filter membrane with 10 kDa nominal cut-off were boiled for 10 min in loading buffer consisting of 50 mM Tris/HCl (pH 6.8), 1% SDS, 1% 2-mercaptoethanol, 0.1% bromophenol blue and 10% glycerol. Portions containing identical amounts of extracted proteins were applied to a 8% tricine/SDS-PAGE gel. The proteins were then electrophoretically separated according to Schägger and Jagow [20] and blotted onto 0.45 μm polyvinylidene fluoride membrane (Millipore), which was blocked with 5% powdered milk in 20 mM Tris/HCl, pH 7.6 (TBS buffer) for 1 hour. The membrane was washed with TBS buffer containing 0.1% Tween-20 and incubated for 1 hour in TBS buffer supplemented with 1% powdered milk and a rabbit polyclonal antibody raised against the HvCKX9 or ZmCKX1 [21]. The membrane was rinsed four times with TBS buffer supplemented with 0.1% Tween-20 then incubated in TBS buffer with 1% powdered milk and anti-rabbit IgG horseradish peroxidase conjugate (Sigma) for 1 hour. The secondary antibody was washed out in the same way as the primary antibody then signals were detected using Amersham ECL Plus Western Blotting Detection Reagents (GE Healthcare).

### Measurement of CK contents

The procedure used for CK purification was a modification of the method described by Faiss [22]. Deuterium-labeled CK internal standards (OlChemIm) were added, each at 1 pmol per sample, to determine CK content using an isotope dilution method [23]. The samples were purified using immunoaffinity chromatography based on wide-range specific monoclonal antibodies against CKs [24]. The samples were purified, concentrated and analyzed with an ultraperformance liquid chromatograph Acquity UPLC coupled to Xevo TQ MS ™ API (Waters) triple quadrupole mass spectrometer equipped with an electrospray interface as previously described in detail [25].

## Results

### *CKX* and *IPT* gene families and their expression profiles

Two *CKX* genes in barley, *HvCKX1* and *HvCKX9* (formerly annotated as *HvCKX2*), have been previously characterized in detail [11]. Examination of expressed sequence tag (EST) collections and full-length cDNA libraries revealed that six other *CKX* genes are expressed in barley tissues (Table S1). Recently, a rough draft of the barley genome covering more than 90% of the expressed genes has been released [26] (The International Barley Genome Sequencing Consortium 2012). Using BLAST with contig data from 454 sequencing (http://webblast.ipk-gatersleben.de/barley/), in total 11 open reading frames that make up the whole *CKX* gene family of barley were defined [27]. Genes coding for two tRNA::IPTs (IPT1 and IPT10) and five adenylate IPTs (IPT2 to IPT5, IPT7) were identified (Table S2, Text S1) by blasting against above mentioned 454 sequencing library, with all characterized maize IPTs as a query. A phylogenetic tree of all maize, rice, barley and Arabidopsis IPT proteins clearly shows that cereal adenylate IPTs cluster together, forming four clades while Arabidopsis enzymes form two other clades (Figure S1). *tRNA::IPT* genes are more conserved among plant species, forming two clades.

**Figure 1:**
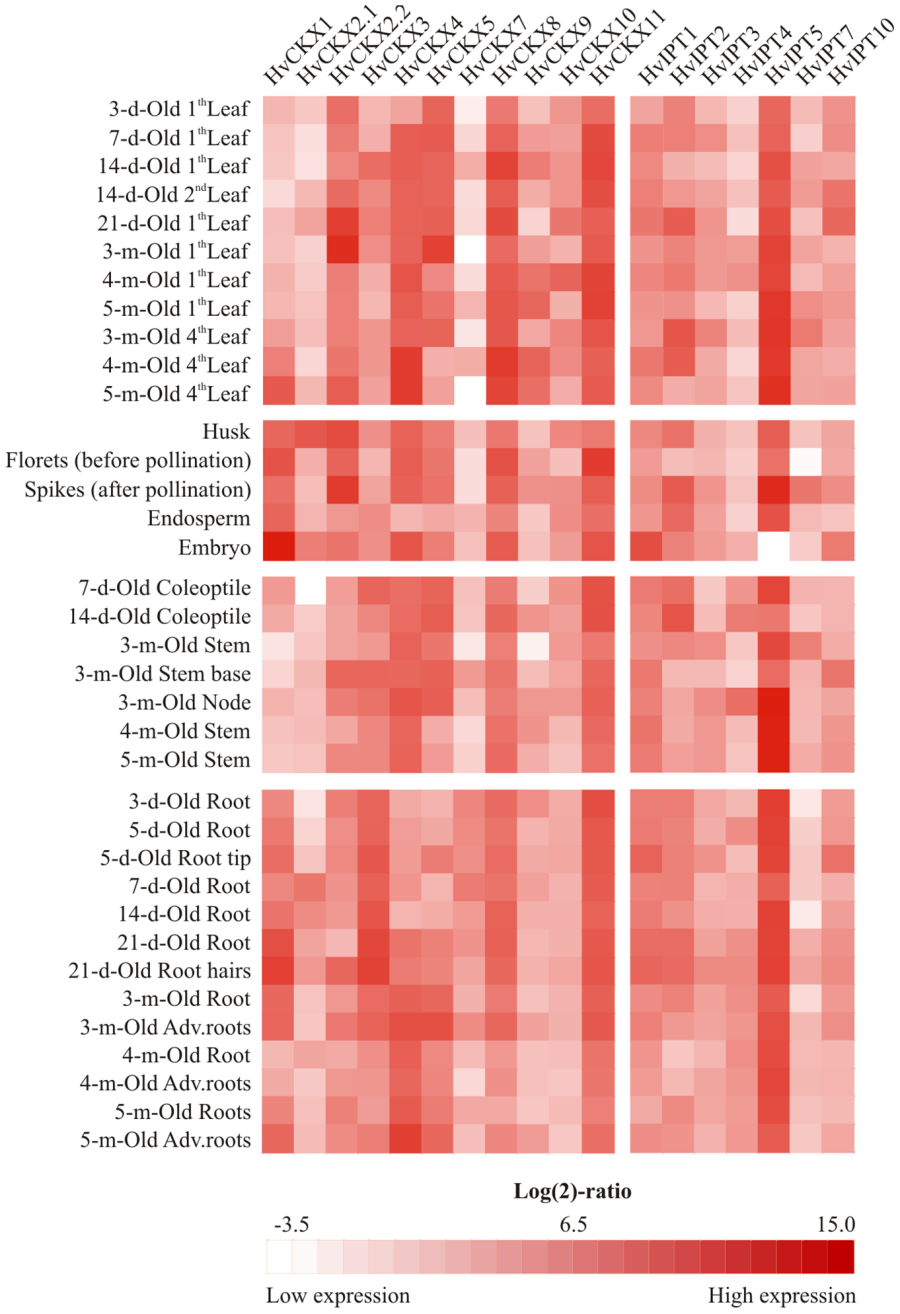
Expression profiles of *CKX* and *IPT* genes in indicated tissues and organs during the lifespan of barley plants. The heatmap illustrates transcript levels detected in 1-ratios and are colour-coded from white to red (the lowest and highest detected transcript numbers, respectively). Real values of at least two biological replicates with standard deviations are summarized in Table S3.

qPCR expression profiling revealed tissue-specific and development stage-related expression patterns for most of the genes (Table S3). *HvCKX1* gene transcripts predominantly accumulated in the embryo and to lower extents in developing ears and, together with *HvCKX3*, seedling roots (Figure 1). *HvCKX4* were the most prevalent *CKX* transcripts in older roots and leaves. The most abundant transcripts in seedling leaves were *HvCKX8* transcripts, which were also abundant in senescent leaves. *HvCKX9* transcripts accumulated in aerial parts, but their levels fluctuated significantly, peaking in newly emerged leaves of the plants in the flowering stage (Figure 1). The highest concentrations of *HvCKX2.2* and *HvCKX5* transcripts were detected in the leaves of 3-month-old plants, while *HvCKX2.2* was also strongly expressed in flowers. *HvCKX2.1* was significantly expressed only in young roots and husks, conflicting with a recent hypothesis that it shares functional redundancy with its closest paralogous gene *HvCKX2.2* [27]. *HvCKX10* was co-expressed with *HvCKX9* in young leaves and contributed to the endosperm and stem *CKX* transcript pool (Figure 1, Table S3). Expression of *HvCKX7* was generally very weak except in young roots. *HvCKX11*, the only *CKX* with cytosolic localization, was stably expressed across all tissues, similar to the reported expression pattern of the maize ortholog *ZmCKX10* [24].

Both *tRNA::IPT* genes showed fairly strong and stable expression patterns, with maxima in young leaves, root tips and embryos and minima in older tissues and endosperm (Figure 1 and Table S3). The only adenylate *IPT* gene that was relatively abundantly expressed in vegetative tissues and reproductive organs was *HvIPT5* (Figure 1), resembling the expression patterns of its closest orthologs *ZmIPT5* and *ZmIPT6*, the only adenylate *IPT* genes that are strongly expressed in maize leaves and roots [24]. Transcript levels of the other four adenylate *IPT* genes were rather low, except for very spatially restricted expression in distinct organs such as fully developed leaves, coleoptiles and spikes (*HvIPT2*), younger stems and spikes (*HvIPT7*) and coleoptiles and nodes (*HvIPT4*). Surprisingly, before pollination developing florets generally have low transcript levels of all *IPT* and *CKX* genes, indicating that CK metabolism is slow in them. *De novo* CK production is likely attenuated in embryos where no *HvIPT5* transcript was detected (Figure 1).

Probes for five *CKX* (*HvCKX1*, *4*, *5*, *8* and *9*) and one *IPT* (*HvIPT1*) genes are present on the 22k Affymetrix Barley Genome Array. The expression potential of selected probes in different organs and tissues is mostly consistent with expression profiles obtained by qPCR (Figure S2) [28].

### Tissue localization of two CKX proteins

**Figure 2:**
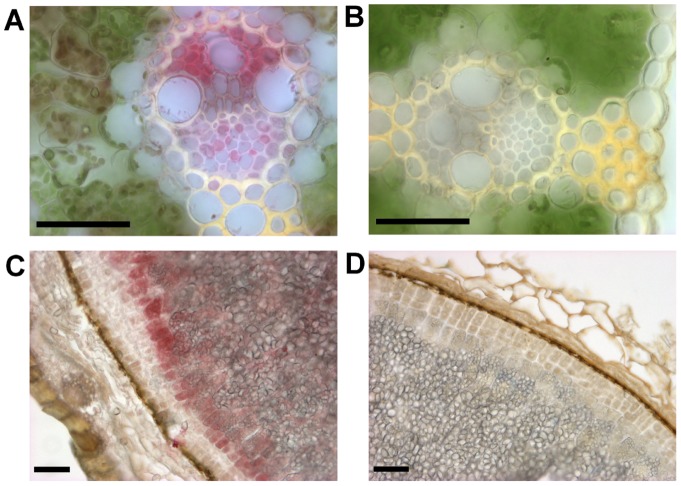
Imunohistochemical localization of barley CKX proteins. Proteins were detected in paraformaldehyde-fixed tissues using antibodies raised against HvCKX9 (**A**) and HvCKX1 (**C**) fragments. Control sections were processed in the same way as samples but omitting incubation with the primary antibody (**BD**). Mature grains in the hard dough stage (**CD**) and 10-day-old first leaves (**AB**) were sectioned on a vibratome. The bar indicates 50 µm.

To identify the precise localization of CKX proteins, two specific polyclonal rabbit antibodies were raised against the most distinct (C-terminal) domains, 139 and 244 amino acids long for HvCKX1 and HvCKX9, respectively. The specificity of the antibodies to bind predominantly the HvCKX1 or HvCKX9 protein targets was tested in assays with equimolar amounts of each recombinant protein fragment. Mutual cross-reactivity was around 10% (Figure S3), but possible cross-reactivity with other HvCKXs, especially those with high homology to HvCKX9, could not be excluded. In addition, false positive staining of other abundant proteins, such as peroxidases, cannot be completely excluded as levels of CKXs are generally low in plant tissues [29]. Anti-HvCKX9 antibody detects CKX proteins predominantly in vascular bundles in leaf sections, mainly in (or around) xylem cells and phloem accompanying cells (Figure 2AB). The most abundant staining with the anti-HvCKX1 antibody was detected in the aleurone layer of filled grains (30 days after pollination) and to a lesser extent in endosperm (Figure 2CD). The signal was also detected in the vasculature of root sections from 14-day-old seedlings, but it was very faint, probably because the vascular cells are significantly larger than embryonic cells. Anti-HvCKX1 signal was not detected in sections from seedling leaves, in accordance with the recorded *HvCKX1* expression profile (Figure 1).

### Constitutive overexpression of CKX in barley

The most strongly limiting factor for genetic engineering of most cereal species is very low transformation efficiency, which is further attenuated by inefficient subsequent selection. However, a novel high-throughput transformation protocol for the model barley variety Golden Promise has been developed recently [17]. Using this protocol, approximately 550 and 950 immature barley embryos of cv. Golden Promise were transformed with a binary vector carrying the subcloned genomic version of *HvCKX9* and intronless maize *ZmCKX1* (the closest orthologous gene to *HvCKX1*, sharing 74.5% identity at the amino acid level), respectively, under control of the ubiquitin maize promoter (*Ubi*). The two constitutively overexpressed enzymes differ in biochemical properties and substrate preferences. While ZmCKX1 is a secreted glycoprotein that is significantly activated in the presence of electron acceptors [12], [21], HvCKX9 has an unpredictable subcellular localization and its *in vitro* activity is considerably lower [11]. ZmCKX1 predominately cleaves free CK bases, whereas HvCKX9 has a higher preference for *N* ^9^-substituted CKs (Table S4).

**Figure 3:**
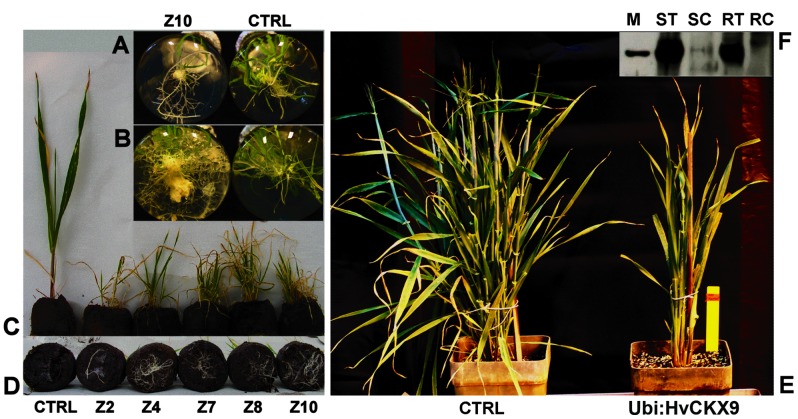
Phenotype of T0-generation barley *Ubi::HvCKX9 and Ubi::ZmCKX1* transformants. Proliferating root systems of *Ubi::ZmCKX1* regenerants in *in vitro* culture 20 and 35 days after transfer to the rooting medium (**A**) and (**B**), respectively. The aerial parts (**C**) and root systems (**D**), respectively, of 65-day-old transformants transferred to soil pellets, CTRL – control plant regenerated *in vitro*, Z2 to Z10– independent transformants with *Ubi::ZmCKX1*. Aerial parts of *Ubi::HvCKX9* plant 5 months after regeneration from callus (**E**). Western blot analysis of *Ubi::HvCKX9* (**F**); M – marker 60 kDa, ST – proteins extracted from *HvCKX9* transformed shoot and roots (RT), and control shoot (SC) and roots (RC).

After the selection with hygromycin, only one plantlet was obtained for the *HvCKX9* construct, and 13 for *ZmCKX1* regenerated from 7 independent calli. Each analyzed *Ubi::ZmCKX1* plant (Z2, Z4, Z7, Z8, Z10) was from an independent callus. When a root system had developed, all plantlets were transferred to soil and confirmed to have high levels of transgene transcripts and elevated CKX activity (Table 1). The total specific activity with iP was 18.2-fold higher in leaves and 1.7-fold higher in roots of the *HvCKX9*-overexpressing plant than in control plants regenerated *in vitro*. When iP9G was used as the substrate, the increases were 18.3 and 4.3-fold in the leaves and roots, respectively. Higher activity in the roots with iP9G confirmed the larger contribution of HvCKX9 isozyme to the total CKX activity since iP9G is the preferred substrate for this isozyme (Table S4), which is not expressed significantly in wild type roots. In *ZmCKX1* overexpressers, 40 to 320-fold increases in the specific activity with iP were measured in extracts from transgenic leaves and 5 to 20- fold increases in the roots. Western blotting with HvCKX9-specific antibodies revealed a significant accumulation of the protein in leaves as well as roots of regenerated *Ubi::HvCKX9* plants (Figure 3F).

**Table 1:** Specific CKX activity in transformed tissues.

Sample	CKX activity (pkat mg^−1^ of protein)	
	iP	IP9G
***Ubi::HvCKX9***		
CTRL – leaves	0.49±0.06	0.65±0.09
CTRL – roots	13.12±2.09	2.56±3.72
G1– leaves	8.92±0.91	11.92±1.81
G1– roots	22.28±2.63	10.97±1.43
***Ubi::ZmCKX1***		
Z2– leaves	20.33±2.42	ND
Z2– roots	69.89±5.85	ND
Z4– leaves	158.70±10.12	ND
Z4– roots	236.95±9.84	ND
***PHT::ZmCKX1 (T0-generation)***		
CTRL – leaves	0.82±0.04	ND
CTRL – roots	19.17±5.42	ND
PHT1– leaves	16.22±2.44	ND
PHT1– roots	190.04±23.42	ND
PHT2– leaves	3.88±0.53	ND
PHT2– roots	168.90±34.33	ND
***PHT::ZmCKX1 (T1-generation)***		
WT – leaves	0.42±0.23	ND
WT – roots	8.64±3.45	ND
T1-PHT2– leaves	2.33±0.97	ND
T1-PHT2– roots	112.10±9.92	ND

Results for two out of seven independent transformants with the *Ubi::ZmCKX1* construct and two out of five with the *PHT::ZmCKX1* construct, selected to represent plantlets yielding the most divergent values, obtained from CKX assays with the preferred substrate for ZmCKX1 (iP) and HvCKX9 (iP9G); ND – not determined; CTRL – non-transformed plant regenerated *in vitro*; WT – plant germinated from wild-type grain.

Regeneration of shoots from calli transformed with the constructs constitutively expressing *CKX* genes was very limited; only one and 13 *Ubi::HvCKX9* and *Ubi::ZmCKX1* plantlets, respectively, rooted under our conditions. All of these plantlets were transferred to soil. The root system of all of these plants massively proliferated following transfer to the rooting medium (Figure 3AB) and soil (Figure 3D). Generally, the growth of the plantlets was very slow and the few leaves that emerged turned yellow and senesced prematurely (Figure 3C). After transfer to a normal-sized pot, surviving plants did not tiller properly (Figure 3E), their culms were shorter than those of wild-type controls and it died without flowering approximately 6 months after the regeneration.

The higher number of regenerants carrying the *Ubi::ZmCKX1* construct, *relative* to *Ubi::HvCKX9*, was probably due to supplementation of the regeneration medium with an artificial CK (BAP), which is hormone-free in the standard procedure [17]. Three regenerated plants were transferred to the rooting medium supplemented with 0.1 μM of a potent CKX inhibitor, 2-chloro-6-(3-methoxyphenyl) aminopurine (INCYDE) [30]. Treatment by the inhibitor was maintained after the plants were transferred to the soil by spraying (1 to 10 μM) or watering (0.1 μM) every third day. The inhibitor-treated plants lived longer than untreated transgenic plants. However, all the plants died after 18 to 42 weeks without reaching the onset of flowering.

### Overexpression of *CKX* under root-specific promoters

In this experiment we cloned promoter sequences of barley genes encoding a phosphate transporter (PHT1-1) [13] and root abundant transcription factor (RAF) [14] and confirmed their ability to drive *GUS* gene expression in Arabidopsis (data not shown). In both cases, no GUS staining was observed in sections of leaf tissues. Barley was transformed in two time-independent experiments with *CKX* constructs driven by these promoters. Following the first round of transformations, three plants transformed with *PHT::ZmCKX1* (PHT-A to PHT-C) and one with *RAF::ZmCKX1* were regenerated. Only two *PHT::ZmCKX1* regenerated plants (PHT-A and PHT-C) yielded any grains (several in both cases).Three PHT-A and three PHT-C T1-generation plants with confirmed transgene integration were cultivated hydroponically and harvested three weeks after germination. Both lines showed significantly increased dry root biomass (PHT-A 58.7±11.5 and PHT-C 42.7±14.3 mg, compared to 26.2±11.2 mg determined from five wild-type plants). Unfortunately, none of the T1-generation plants yielded grains with an integrated transgene.

**Figure 4:**
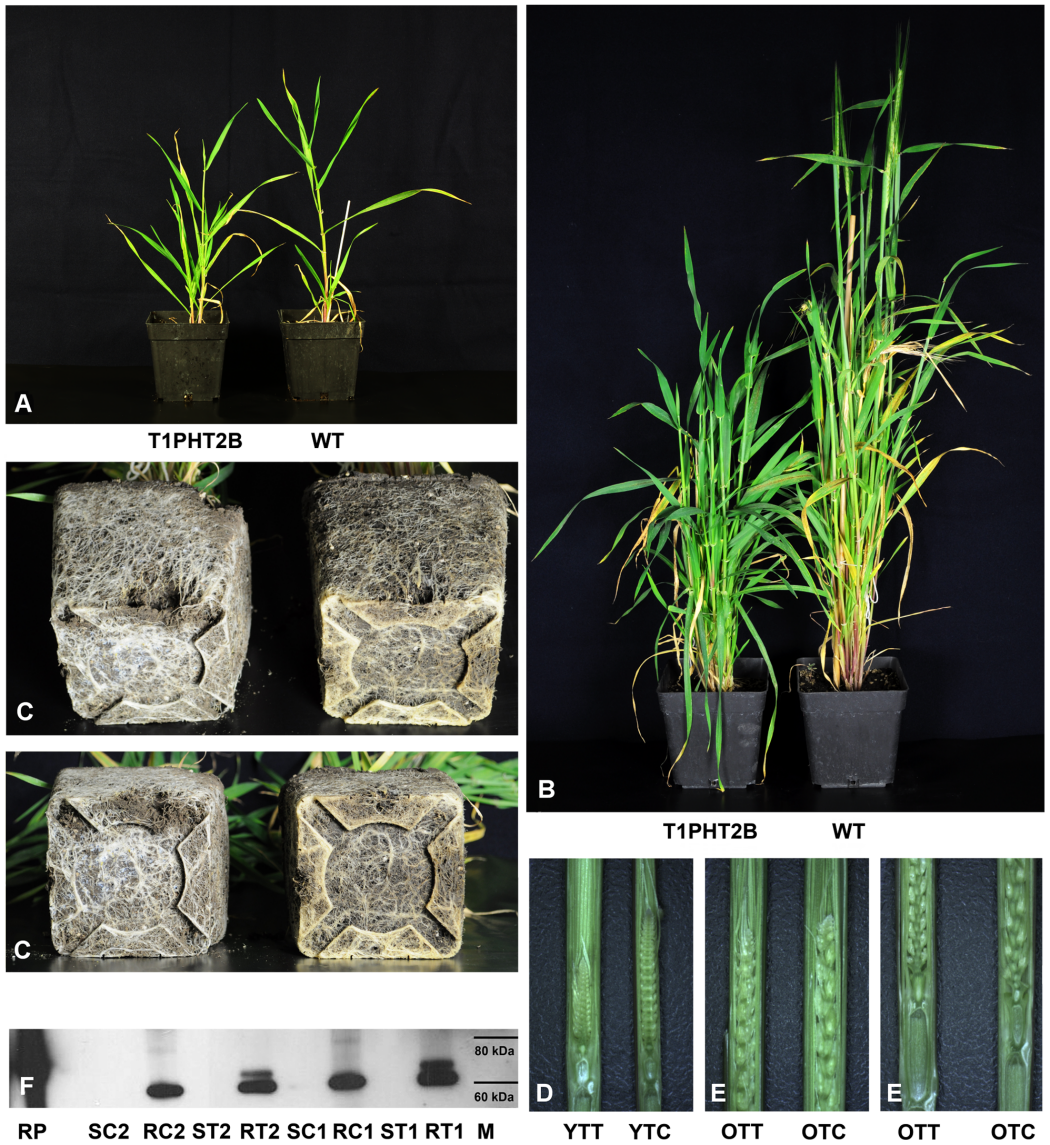
Phenotype of T1-generation *PHT::ZmCKX1* barley transformants. 2-month-old aerial parts of T1-generation plant (**A**), 4-month-old aerial parts (**B**) and its root system (**C**); WT – non-transformed plant. Developing flowers exposed from the flag sheath of the youngest (**D**) and the oldest tillers (**E**) of T1 transgenic plants (left, YTT and OTT) and appropriate control plants (right, YTC and OTC). Western blot analysis of two independent 4-month-old T1 PHT2 plants (**F**); M – marker 60 kDa and 80 kDa, RP – ZmCKX1 recombinant protein [21], proteins extracted from transgenic (ST) and control (SC) stems and roots (RT, RC).

**Figure 5:**
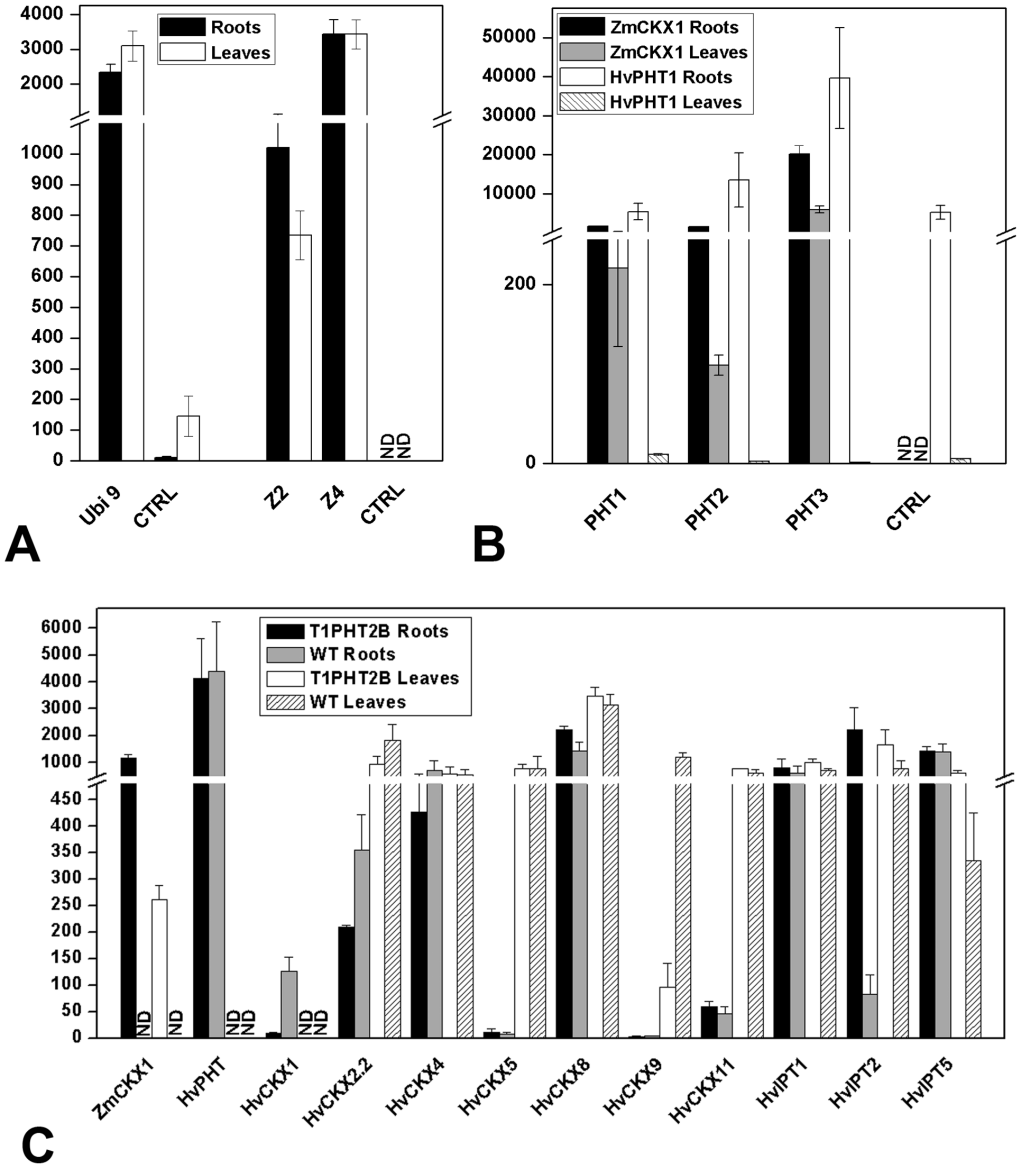
Transcript abundance of the transgene in leaves and roots of T0-generation plants transformed with *ZmCKX1* (Z2, Z4) or *HvCKX9* (Ubi 9) genes under control of the *Ubi* promoter (A). Transcript abundance of the transgene and *PHT1-1* gene in leaves and roots of T0-generation plants transformed with *ZmCKX1* under control of the *PHT1-1* promoter (**B**). Transcript abundance of the transgene, *PHT1-1* gene and the most abundantly expressed endogenous *CKX* and *IPT* genes in leaves and roots of T1-generation plants transformed with *ZmCKX1* under control of the *PHT1-1* promoter (PHT2 line) (**C**). Abundance expressed as transcript number per ng of total RNA amplified by qPCR with respect to primer pair efficiency. RNA from two biological replicates was transcribed in two independent reactions, and PCR was performed in duplicate. Mean values with standard deviations are shown.

In the second experiment, 300 immature embryos with each construct were transformed. In total, 10 *PHT::ZmCKX1* (PHT1 to PHT5) and three *RAF::ZmCKX1* transgenic plants regenerated from five and two independent calli, respectively, and were studied in more detail. As before, plants transformed with each construct formed more robust root systems than control plants (Figure 4C, Figure S4C). Aerial parts of these plants grew slowly shortly after transfer to soil, but in contrast to *Ubi::ZmCKX1* plants they did not senesce prematurely (Figure 4AB, Figure S4AB). In addition, onset of flowering was significantly delayed relative to its timing in control plants (2 to 3 months) or not observed at all. During the prolonged vegetative period *PHT::ZmCKX1* lines tended to form more new tillers. Generally, transgenic lines formed less fully open ears than control plants, many ears stayed closed in the leaf sheath, or no ears were produced (all *RAF::ZmCKX1* and PHT3 plants). Levels of transgene expression corresponded to the strength of the observed phenotypic deviations, notably *ZmCKX1* expression was two orders of magnitude lower in PHT2 plants (Figure 5), which yielded viable grains, than in PHT3 plants, which did not form any ears. T1-generation PHT2 plants sown in soil showed the same morphological alterations as those described for the T0-generation (Figure 4, Figure S4).

Higher production of ZmCKX protein in roots than in stems was confirmed by Western blotting with anti-ZmCKX1 antibody (Figure 4F). While no signal was detected in stem samples, a novel protein band of 65 kDa, corresponding to ZmCKX1, was observed in PAGE analysis of protein extracts from transgenic roots, which was not detected in analysis of extracts of control plants. The thick band of 60 kDa detected in extracts of both transgenic and control roots can be attributed to unspecific interaction of the antibody to some abundant endogenous CKX. Nevertheless, the *ZmCKX1* transgene was significantly more strongly expressed in leaf samples collected throughout the vegetative period of T0 and T1 plants than the *PHT1-1* gene, indicating that the region of the *PHT1-1* promoter used to express the *ZmCKX1* gene lost its strict specificity to root tissues (Figure 5). The integration site of the transformation cassette may also contribute to the level of transgene expression. One out of three independent T0 *PHT::ZmCKX1* transformants showed comparable expression of the transgene in roots and leaves, whereas the other two were expressed an order of magnitude more weakly in leaves. Accordingly, only T0 transformants of line PHT2, in which expression under the recombinant promoter was weakest (but still two orders of magnitude stronger than under the endogenous *PHT1-1* promoter), eventually yielded a few grains. Although T1 plants of line PHT2 formed many open ears, the great majority of them were empty. In total we obtained five grains (T2-generation), but in all of them the transgene was segregated out. These findings indicate that CK homeostasis was also probably disturbed during gamete formation or maturation.

### Alteration of CK levels in transgenic barley

CKs were quantified in transgenic lines from which more than three plants were regenerated (T0 *Ubi::ZmCKX1*, T0 and T1 *PHT::ZmCKX1*). Four types of isoprenoid CKs, namely *trans*-zeatin (tZ), *cis-*zeatin (cZ), dihydrozeatin (DHZ) and iP, had detectable levels of free bases and various sugar conjugates in barley tissues. Generally, the dominant CK form was cZ and its derivatives followed by tZ and iP. Levels of DHZ and its derivatives, in contrast to the other CKs, were rather low.

In T0 plants, as expected, levels of most measured CK forms were lower than in control plants, due to the increase in CKX activity (Table S5). Only levels of iP, cZ, tZ riboside and tZ-9-glucoside were significantly elevated in the transgenic roots of *Ubi::ZmCKX1* plants, whereas the other analyzed line *PHT::ZmCKX1* showed increases in tZ and isopentenyladenosine-5′-monophosphate. The observed inconsistency was not surprising, as all the data were obtained from T0-plants, which passed through *in vitro* regeneration, and development was significantly retarded in the transgenic lines.

Therefore, in more detailed analysis we focused on T1 *PHT::ZmCKX1* plants. Several samples from aerial parts at different developmental stages were collected from both control and transgenic 4-month-old plants: leaves from the youngest tiller 14 days after emergence from the crown, fully developed leaves from secondary tillers (approx. 1-month-old), and mature leaves from the first tiller (4-months-old). As expected, levels of all CKs, except iP, were significantly lower, relative to wild type levels, in the two young leaf samples (Table 2). However, the oldest transgenic leaves accumulated significantly higher amounts of CKs, especially those considered the most active: tZ, iP and their ribosides. Surprisingly, levels of all four CK nucleotides, regarded as primary biosynthetic products, were also uniformly elevated. Similar patterns were observed in transgenic roots, where only the tissue from the bottom of the 4-month-old root system was collected, due to limitations imposed by the cultivation conditions. To distinguish possible artifacts associated with developmental delay of the transgenic line, roots from younger (3-month-old) control plants were also analyzed. In transgenic roots, levels of active tZ and tZ riboside were higher than in both types of control samples. The iP concentration fluctuated more in control plants, being higher in younger and lower in older roots than in transgenics. Nevertheless, levels of iP, tZ and cZ nucleotides were higher in transgenic roots, indicating that older transgenic roots reinforced CK biosynthesis. In transgenic roots, the only manifestations of depleted levels of active CKs in younger roots were greatly diminished levels of CK-9-glucosides, considered to be irreversible deactivation products of CK metabolism that accumulate with the age of the plant (Table 2).

**Table 2:** Endogenous CK levels (pmol g^−1^ fresh weight) in leaves and roots of T1 *PHT::ZmCKX1* barley plants.

	PHT2	WT	WT	PHT2	WT	PHT2	WT	PHT2	WT
	4-m-Old Roots	3-m-Old Roots	4-m-Old Roots	14-d-Old Leaves	14-d-Old Leaves	1-m-Old Leaves	1-m-Old Leaves	4-m-Old Leaves	4-m-Old Leaves
tZ	*0.67±0.01	0.54±0.03	0.45±0.02	*1.67±0.25	2.52±0.31	*1.29±0.07	2.40±0.34	*2.80±0.25	1.69±0.28
tZR	*1.36±0.25	0.78±0.08	0.82±0.12	*0.63±0.15	0.99±0.16	*0.56±0.06	1.53±0.12	*0.25±0.03	0.10±0.01
tZOG	0.30±0.04	0.40±0.02	0.41±0.08	*4.65±0.42	6.97±0.55	6.08±1.19	8.14±0.76	*3.53±0.40	2.32±0.38
tZROG	*0.23±0.02	0.15±0.01	0.15±0.01	*0.10±0.02	0.16±0.04	*0.15±0.02	0.27±0.09	0.11±0.01	0.12±0.01
tZ9G	*3.08±0.84	11.04±1.61	11.23±0.79	*23.58±3.42	44.48±2.82	*27.81±4.53	54.62±6.95	41.65±4.07	35.18±4.93
tZR5′MP	*2.61±0.39	0.69±0.05	0.66±0.06	*0.61±0.24	1.19±0.24	*0.80±0.10	1.69±0.38	*0.53±0.07	0.29±0.05
Active	2.03	1.32	1.27	2.30	3.51	1.85	3.93	3.05	1.79
Total	8.25	13.60	13.72	31.24	56.31	36.69	68.65	48.87	39.70
cZ	0.22±0.02	0.26±0.03	0.28±0.05	*0.29±0.03	0.42±0.05	0.30±0.02	0.28±0.02	1.10±0.20	1.45±0.13
cZR	2.49±0.26	3.05±0.24	3.17±0.42	*1.53±0.31	2.30±0.28	1.35±0.14	1.74±0.28	1.05±0.22	1.20±0.13
cZOG	*1.90±0.17	3.43±0.42	3.20±0.24	*13.23±1.49	18.36±0.37	15.28±0.82	12.32±1.56	*32.12±4.76	22.27±2.68
cZROG	5.56±0.31	5.21±0.66	5.81±0.63	1.56±0.14	1.99±0.14	2.07±0.20	1.77±0.25	1.76±0.04	1.68±0.18
cZ9G	*0.02±0.00	0.41±0.02	0.45±0.08	*0.18±0.03	0.32±0.05	*0.24±0.04	0.38±0.03	0.82±0.09	0.92±0.19
cZR5′MP	8.42±0.79	5.95±0.93	7.22±1.37	*6.11±0.56	14.78±2.04	*3.56±0.14	8.36±1.18	*2.85±0.44	6.25±0.85
Active	2.71	3.31	3.45	1.82	2.72	1.65	2.02	2.15	2.65
Total	18.61	18.31	20.13	22.90	38.17	22.80	24.85	39.70	33.77
DHZ	UDL	UDL	UDL	UDL	UDL	UDL	UDL	UDL	UDL
DHZR	0.03±0.00	0.03±0.01	0.03±0.00	UDL	UDL	UDL	UDL	UDL	UDL
DHZOG	0.37±0.05	0.39±0.05	0.20±0.03	*1.65±0.40	3.14±0.41	*1.71±0.22	3.68±0.44	4.37±0.51	4.12±0.42
DHZROG	1.66±0.21	1.11±0.13	1.09±0.13	*0.24±0.05	*0.71±0.09	0.49±0.07	*0.85±0.04	*0.40±0.03	0.74±0.04
DHZ9G	UDL	UDL	UDL	UDL	UDL	UDL	UDL	UDL	UDL
DHZR5′MP	UDL	UDL	UDL	UDL	UDL	UDL	UDL	UDL	UDL
Total	2.06	1.53	1.32	1.89	3.85	2.20	4.53	4.77	4.86
iP	*0.32±0.03	0.70±0.01	0.10±0.01	0.21±0.03	0.22±0.03	0.32±0.05	0.29±0.05	0.25±0.04	0.17±0.03
iPR	2.91±0.21	3.54±0.32	2.25±0.32	*2.27±0.35	3.31±0.08	2.38±0.26	2.47±0.26	*3.52±0.23	2.48±0.38
iP9G	*0.25±0.04	2.03±0.07	1.40±0.04	*0.39±0.06	0.91±0.15	*0.35±0.06	0.93±0.16	*4.71±0.74	1.93±0.23
iPR5′MP	*5.54±0.38	2.90±0.40	2.09±0.06	*1.60±0.15	2.74±0.09	*2.18±0.16	2.83±0.18	*2.12±2.72	0.65±0.90
Active	3.23	4.24	2.35	2.48	3.53	2.70	2.76	3.77	2.65
Total	9.02	9.17	5.84	4.47	7.18	5.23	6.52	10.60	5.23

The CKs analyzed included: free bases of *trans*-zeatin (tZ), *cis*-zeatin (cZ), dihydrozeatin (DHZ) and *N* ^6^-isopentenyladenine (iP); their N^9^-ribosides (tZR, cZR, DHZR, iPR); *O*-glucosides (tZOG, cZOG, DHZOG); *O*-glucoside-*N* ^9^-ribosides (tZROG, cZROG, DHZROG); *N* ^9^-glucosides (tZ9G, cZ9G, DHZ9G, iP9G); and *N* ^9^-riboside-5′-monophosphates (tZR5′MP, cZR5′MP, DHZ5′MP, iPR5′MP); sums of free bases and *N* ^9^-ribosides are considered to comprise the pool of active CKs; mean values with standard deviations obtained from three biological replicates are presented; asterisks indicate significant differences between control and transformed tissue according to Student's unpaired t-tests at P≤0.05; UDL – under detection limit; WT – plants germinated from wild-type grains.

## Discussion

Knowledge of the full genome sequence of a species is essential for understanding its natural genetic variation and developing modern breeding strategies. Recently, novel high-throughput sequencing technologies have been exploited to unravel the structure of the barley genome [26]. A systematic synteny analysis with model species from the Poaceae family with already annotated genomes (rice, maize, sorghum and *Brachypodium*) confirmed the existence of over 30,000 barley genes. Hence, we were able to find sequences with high homology to all characterized maize and rice CKXs and IPTs, indicating that CK homeostasis is regulated in a similar manner in all Poaceae subfamilies. Almost identical expression profiles of the closest orthologous genes in barley and maize organs support this hypothesis (Figure 1; [24]). The barley genome appears to have relatively low complexity as there is only one paralogous gene in most clades of the IPT phylogenetic tree (Figure S1), in contrast to the maize and rice genomes, where almost all genes in separated clades are duplicated. Barley also appears to possess relatively few members of other CK-related gene families. For instance, only three and seven annotated gene structures encoding CK receptors and CK type-A response regulators, respectively, were found in the latest version of the barley genome annotation (August 2012) compared to 11 and 21, respectively, in the maize genome [31]. Similarly, the barley genome encodes only seven CK-specific phosphoribohydrolases (LOGs), compared to 11 identified orthologs in rice [32]. Although LOG activity contributes considerably to the active CK pool in plant tissues by converting non-active CK nucleotides to active free bases, the rate-limiting step of CK production and turnover rates is biosynthesis, catalyzed by IPT. Further, in contrast to *IPT* and *CKX*, the expression of maize *LOG* genes does not reportedly clearly respond to the perturbation of CK homeostasis by exogenous applications of CK [33].

Regulation of endogenous CK contents in a precise spatial manner enables the development of novel crop species with higher yields or greater tolerance to various stresses [34]. For instance, Werner and colleagues demonstrated that root-driven overexpression of *CKX* genes can increase the root biomass of model dicot plants by 40% [35], without any significant detectable changes in shoot development or productivity. Moreover, the transgenic plants the cited authors examined had higher survival rates after severe drought treatment and accumulated higher amounts of several chemical elements in their shoots. Recently, endogenous CK contents in barley plants have been altered by silencing the *HvCKX1* gene, which resulted in higher grain filling [36]. This finding can be explained by reduced activity of this key CK degradation enzyme in the regulatory aleurone layer, where the product of the *HvCKX1* gene strongly accumulated (Figure 2C), leading to increased concentrations of CKs. CKs regulate sink strength by activating cell wall invertases and hexose transporters [37]. Thus, the accelerated mobilization and flow of sucrose could enhance starch accumulation in the endosperm and hence grain filling.

Here, for the first time, we show the consequences of *CKX* overexpression in monocot plants. A highly-efficient germ-line transformation method utilizing a supervirulent *Agrobacterium* strain was applied to obtain stable transgenic lines. We obtained approximately 30% transformation efficiency when an empty vector or vector with no *CKX* gene was integrated into the barley genome, but far fewer regenerated plants overexpressing *CKX* genes. Overall efficiency was below 1% with three out of four constructs used, and only 3% for the other (*PHT::ZmCKX1*). Thus, the transgene activity probably disrupts the hormonal balance, which is essential for the transition to the first leaf primordia in undifferentiated callus tissue. The observed effects of enhanced CKX activity on normal shoot regeneration from transformed callus tissue supports findings of a study where *ZmCKX1* was used as a negative selector gene during soybean transformation [38]. By incorporating the *ZmCKX1* gene under the control of a constitutive promoter into the non-integrated part of the vector, the cited authors significantly reduced numbers of regenerated plants bearing parts of a binary vector backbone.

We have shown that ubiquitous *CKX* overexpression induces CK-deficiency in barley plants, leading to similar morphological alterations to those observed in model Arabidopsis and tobacco plants [6], [8], including distinctively enlarged root systems and retarded development of aerial parts. However, it had a more profound impact on viability than in Arabidopsis and tobacco, as the barley plants did not transit to the reproductive stage and their leaves senesced prematurely. In sharp contrast, onset of senescence was not advanced in any Arabidopsis overexpressor lines examined by Werner and colleagues, indeed their leaves stayed green even longer and they produced larger seeds (but significantly fewer) than those of wild-type controls [6]. None of 14 barley *Ubi::CKX* transformants yielded grains, thus characterization of ubiquitous CK-deficiency in barley plants in more detail was impossible.

Transgenic plants with the *CKX* transgene under control of *PHT1-1* and *RAF* promoters, regulating the expression of genes specifically expressed only in root tissues, were subsequently regenerated. Nevertheless, negative effects on the aerial parts were again visible shortly after their transfer to soil. The *ZmCKX1* transgene was significantly expressed not only in roots, but also in leaves a month after the transfer, although *PHT1-1* and *RAF* transcripts were barely detectable in the same material (Figure 5). This clearly demonstrates that the chosen promoter regions lose their spatial specificity when integrated randomly into the barley genome. An identical promoter sequence of *PHT1-1* was previously found to maintain unambiguous specificity to root tissues in rice plants [12]. Thus, the presence of unknown *cis*-elements upstream of the cloned promoter region seems to be essential to control the root specificity of expression in barley. *Cis*-elements acting as repressors of unspecific expression were shown to be important to keep strict specificity to original tissues in barley transformants [39], [40]. Reduced CK levels in young tillers of T1 PHT2 transformants do not have a negative effect on the formation of inflorescence meristems and flower primordia (Figure 4DE), but the inability to form grains was probably due to low viability and/or pollen production. Other possibility could be insufficiency of pollinated florets to pass into grain filling stage. Both processes have been shown to be sensitive to reduced CK levels [41], [42].

However, in older tissues of T1-generation *PHT::ZmCKX1* transgenic barley plants the diminution of CK levels induced by *ZmCKX1* overexpression caused significant down-regulation of several endogenous *CKX* genes (*HvCKX1, HvCKX9*) and strong up-regulation of *de novo* biosynthesis genes (*HvIPT2*, Figure 5 and Table S6). Hence, the transgenic plants clearly tend to restore the hormonal balance by accumulating higher levels of active CKs in older roots as well as leaves. The existence of such regulatory feedback mechanisms is supported by the finding that *ZmCKX1* expression under the control of a recombinant *PHT1-1* promoter slightly decreased with increasing age of the plants, after peaking in 8-week-old (T0-generation) seedlings (Table S6). Thus, the relatively weak *CKX* expression under the recombinant *PHT1-1* promoter relative to its expression under the strong ubiquitin promoter allowed the plants to acclimatize to primary CK diminution, recover and even prolong their vegetative growth stage and form more tillers, whereas all *Ubi::CKX* plants died prematurely. In fact, the lifespan of T1 *PHT::ZmCKX1* plants (more than 1 year) was significantly longer than the lifespan of a standard spring barley (8 months in a controlled environment), which can be attributed to later accumulation of active CKs. However, it is still not clear if the observed enhancement of root systems of older plants (Figure 4C), in which active CK levels have already increased, is due to enhanced root proliferation in earlier developmental phases or more extensive tillering of the transgenic plants and delayed onset of flowering compared to control plants of the same age.

Another possible explanation takes into account recent findings that a CK-receptor interaction occurs predominantly in the endoplasmic reticulum lumen [43], [44]. Initial CK depletion leads to compensation by enhanced biosynthesis and down-regulation of some endogenous CKXs, but recombinant ZmCKX1, acting mainly in the endoplasmic reticulum, causes persisting local diminution of CKs in this compartment. Hence, the elevated CK content, which can be attributed to high levels in compartments other than the endoplasmic reticulum, is still not sufficient to restore wild-type CK sensing via histidine kinase receptors so plants maintain the-CK deficient phenotype.

In a recent study, manipulations of endogenous CK contents in barley demonstrated the potential utility of *CKX* transgenes in genetic breeding of cereals due to the ability they confer to influence various agronomically important traits [36]. In the present study, we successfully prepared transgenic barley plants with distinctively changed organ proportions in favor of the root system. The alteration in root morphology could be beneficial for growth at sites affected by environmental stresses. For instance, plants with enhanced root systems would be more tolerant of water deficiency during drought periods, and greater branching of roots could improve the anchorage in soil and nutrient uptake of plants with weak and superficial root systems, such as barley. Nevertheless, identification, precise selection and testing of promoter regions that steadily drive expression specifically in the root tissue during ontogenesis and without leakage to other organs is required to further evaluate and rigorously apply this approach to agronomically important genotypes.

## Supporting Information

Figure S1 **Phylogenetic tree of all HvIPT, OsIPT, ZmIPT and AtIPT proteins.** The Phylogram was calculated using the Maximum Likelihood method as implemented in MEGA5.1 software (Tamura *et al*., 2011) based on ClustalW alignment using the Gonnet matrix. Bar = 0.2 amino acid substitutions per site.
(TIF)

Figure S2 Expression profiles of selected *CKXs*, *HvIPT1*, *HvRAF*, *HvACT* and *HvEF1* in indicated barley tissues and organs (A) and during indicated developmental stages (B). Data were generated by Genevestigator software [28]. The intensity of the blue color indicates the strength of *CKX* expression in particular tissues/organs (following subtraction of intensities observed from 806 independent Hv_22k Barley Genome 22k chips processed with RNA extracted from various cultivars of wild type barley).
(TIF)

Figure S3 **Cross-reactivity of antibodies against HvCKX1 and HvCKX9.** Cross-reactivity was determined by Western blotting with an extract of *Escherichia coli* transformed with empty pCRT7/NT-TOPO vector and recombinant protein fragments (all purified by passage through Ni-NTA Sepharose HP). M – marker; lanes 1 and 7– *E. coli* extract (10 ng); lanes 2 and 8– recombinant HvCKX9 fragment (2 ng); lane 6– recombinant HvCKX9 fragment (0.2 ng); lanes 3 and 4– recombinant HvCKX1 fragment (2 ng); lane 5– recombinant HvCKX1 fragment (0.2 ng); left part of the membrane stained with the anti-HvCKX9 antibody and right part with the anti-HvCKX1 antibody.
(TIF)

Figure S4 **Phenotype of T0-generation** ***PHT::ZmCKX1*** **barley transformants.** Aerial parts of two independent transformants sprayed regularly with the CKX inhibitor INCYDE (A) and two independent non-sprayed transformants (B) and their root system (C) 6 months after transfer to soil from *in vitro* culture; PHT1 to PHT3– transgenic lines regenerated from independent calli, A and B – independent plants regenerated from a single callus, CTRL – non-transformed plant regenerated *in vitro*.
(TIF)

Table S1 **Sequences of primers and Taqman probes used for qPCR.** The closest orthologous rice and maize genes together with GenBank accession numbers, references and probe names on the Barley Affymetrix chip are listed.
(DOCX)

Table S2 **Sequences of primers and Taqman probes used for qPCR.** Contig numbers containing ORFs of assigned putative genes with indicated numbers and positions (lengths) of exons, the closest rice and maize orthologous genes together with GenBank accession numbers or previously described ESTs are listed.
(DOCX)

Table S3 **Transcript abundance in indicated barley tissues.** Abundance is expressed as number of transcripts per ng of total RNA amplified by qPCR with respect to primer pair efficiency. RNA from two biological replicates was transcribed in two independent reactions, and PCR was performed in duplicate. Mean values ± standard deviations are shown.
(DOCX)

Table S4 **Relative substrate specificity of ZmCKX1 and HvCKX9.** Activity was measured as described in the ***Materials and Methods*** section (100 mM McIlvaine buffer, pH 6.0, with 0.5 mM 2,3-dimetoxy-5-methyl-1,4-benzoquinone as an electron acceptor and 0.25 mM substrate) with recombinant ZmCKX1 prepared in *Pichia pastoris* [21] and extract from tobacco constitutively overexpressing HvCKX9 [11]. Specific activity with iP considered as 100% was 752 nkat mg^−1^ for ZmCKX1 and 4.2 pkat mg^−1^ for HvCKX9.
(DOCX)

Table S5 **Endogenous CK levels (pmol g^−1^ FW) in leaves and roots of T0 barley plants transformed with** ***Ubi::ZmCKX1*** **and** ***PHT::ZmCKX1*** **.** The analyzed CKs included: free bases of *trans*-zeatin (tZ), *cis*-zeatin (cZ), dihydrozeatin (DHZ) and isopentenyladenine (iP); their N^9^-ribosides (tZR, cZR, DHZR, iPR); O-glucosides (tZOG, cZOG, DHZOG); O-glucoside-N^9^-ribosides (tZROG, cZROG, DHZROG); N^9^-glucosides (tZ9G, cZ9G, DHZ9G, iP9G); and N^9^-riboside-5′-monophosphates (tZR5′MP, cZR5′MP, DHZ5′MP, iPR5′MP). Sum of free bases and N^9^-ribosides are considered to comprise the pool of active CKs. Mean values ± standard deviations from two samples derived from three pooled leaves and cut roots from the bottom of the root system are presented.
(DOCX)

Table S6 **Transcript abundance of barley** ***CKX*** **and** ***IPT*** **gene families in T0-generation** ***PHT::ZmCKX1*** **and control plants regenerated** ***in vitro*** **.** Abundance is expressed as number of transcripts per ng of total RNA amplified by qPCR with respect to primer pair efficiency. RNA from two biological replicates was transcribed in two independent reactions, and PCR was performed in duplicate. Mean values ± standard deviations are shown.
(DOCX)

Text S1 **DNA genomic sequences of predicted** ***HvIPT*** **genes generated from a rough draft of the barley genome.** Shaded sequences indicate start and stop codons, while intron sequences are underlined.
(DOCX)
