# Supplementary figures and images for: Overexpression of Cytokinin Dehydrogenase Genes in Barley (Hordeum vulgare cv. Golden Promise) Fundamentally Affects Morphology and Fertility
Source: PLoS One. 2013 Nov 15;8(11):e79029. doi: 10.1371/journal.pone.0079029 (PMC3829838; doi:10.1371/journal.pone.0079029)

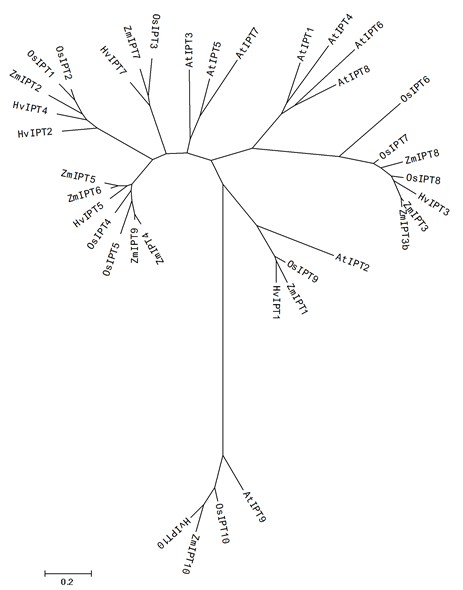

Supplement: Figure S1 — Phylogenetic tree of all HvIPT, OsIPT, ZmIPT and AtIPT proteins. The Phylogram was calculated using the Maximum Likelihood method as implemented in MEGA5.1 software (Tamura et al., 2011) based on ClustalW alignment using the Gonnet matrix. Bar = 0.2 amino acid substitutions per site. (TIF) [file pone.0079029.s001.tif]

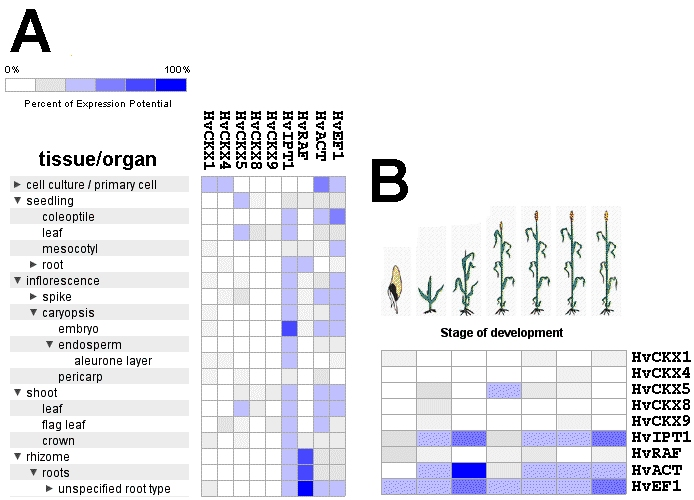

Supplement: Figure S2 — Expression profiles of selected CKXs, HvIPT1, HvRAF, HvACT and HvEF1 in indicated barley tissues and organs (A) and during indicated developmental stages (B). Data were generated by Genevestigator software [28]. The intensity of the blue color indicates the strength of CKX expression in particular tissues/organs (following subtraction of intensities observed from 806 independent Hv_22k Barley Genome 22k chips processed with RNA extracted from various cultivars of wild type barley). (TIF) [file pone.0079029.s002.tif]

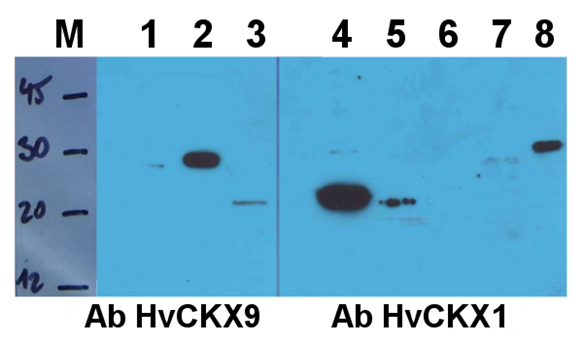

Supplement: Figure S3 — Cross-reactivity of antibodies against HvCKX1 and HvCKX9. Cross-reactivity was determined by Western blotting with an extract of Escherichia coli transformed with empty pCRT7/NT-TOPO vector and recombinant protein fragments (all purified by passage through Ni-NTA Sepharose HP). M – marker; lanes 1 and 7– E. coli extract (10 ng); lanes 2 and 8– recombinant HvCKX9 fragment (2 ng); lane 6– recombinant HvCKX9 fragment (0.2 ng); lanes 3 and 4– recombinant HvCKX1 fragment (2 ng); lane 5– recombinant HvCKX1 fragment (0.2 ng); left part of the membrane stained with the anti-HvCKX9 antibody and right part with the anti-HvCKX1 antibody. (TIF) [file pone.0079029.s003.tif]

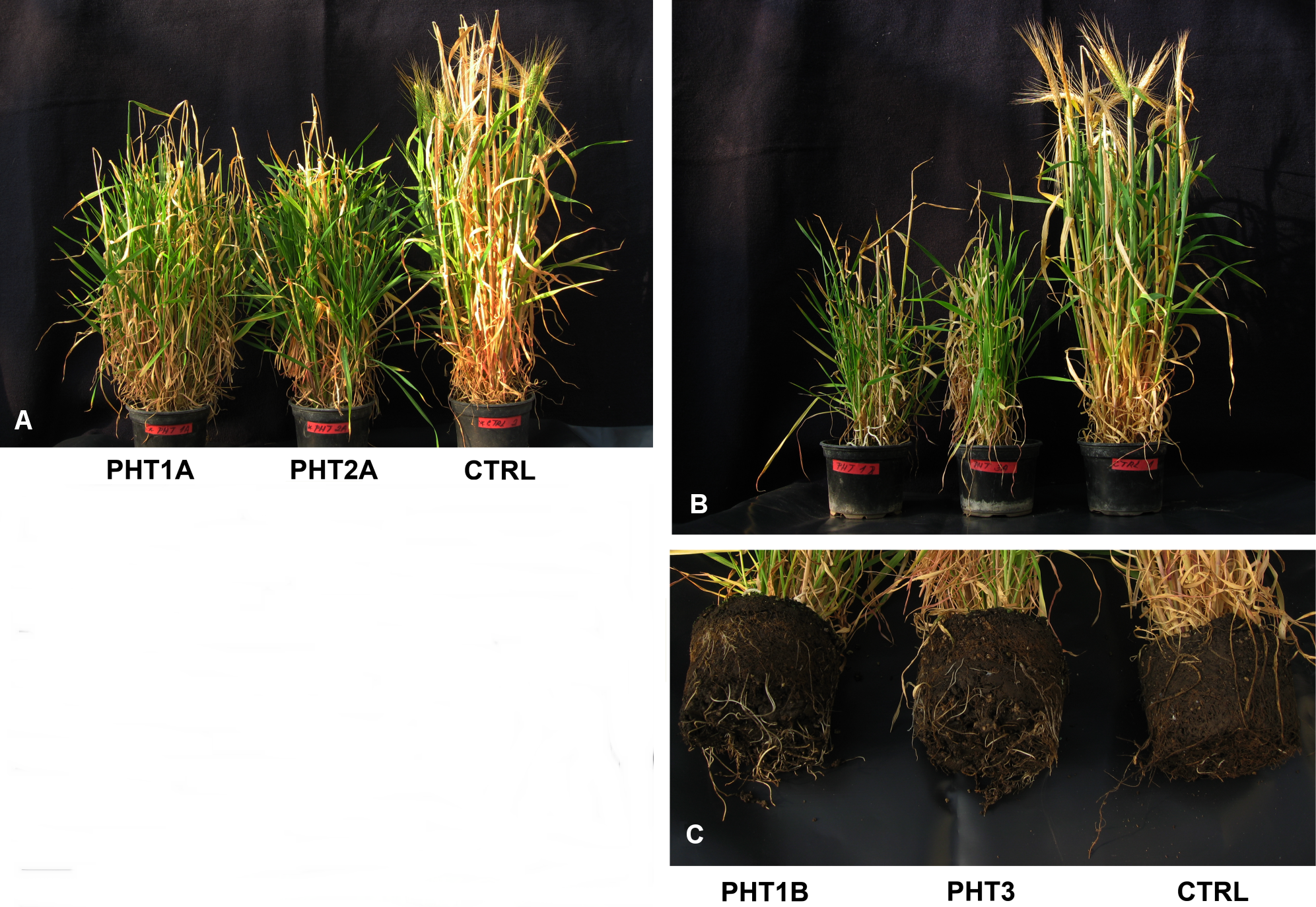

Supplement: Figure S4 — Phenotype of T0-generation PHT::ZmCKX1 barley transformants. Aerial parts of two independent transformants sprayed regularly with the CKX inhibitor INCYDE (A) and two independent non-sprayed transformants (B) and their root system (C) 6 months after transfer to soil from in vitro culture; PHT1 to PHT3– transgenic lines regenerated from independent calli, A and B – independent plants regenerated from a single callus, CTRL – non-transformed plant regenerated in vitro. (TIF) [file pone.0079029.s004.tif]
