# Supplementary material for: Overexpression of Cytokinin Dehydrogenase Genes in Barley (Hordeum vulgare cv. Golden Promise) Fundamentally Affects Morphology and Fertility
Source: PLoS One. 2013 Nov 15;8(11):e79029. doi: 10.1371/journal.pone.0079029 (PMC3829838; doi:10.1371/journal.pone.0079029)
Supplement: Table S1 — Sequences of primers and Taqman probes used for qPCR. The closest orthologous rice and maize genes together with GenBank accession numbers, references and probe names on the Barley Affymetrix chip are listed. (DOCX) [file pone.0079029.s005.docx]

**Table S1. Sequences of primers and Taqman probes used for qPCR.**

| Gene | Sequence of forward and reverse primers (and Taqman probe) | GenBank # | Probe on Barley Affymetrix chip | The closest ortholog | Reference |
| --- | --- | --- | --- | --- | --- |
| HvCKX1 | 5´-TGTGGACAGTAACACAGCAGTTTAAC-3´ | AF362472 | Contig16024_at | OsCKX1/ZmCKX1 | Galuszka *et al*., 2004 |
|  | 5´-CCGTGCCACCTACTATCAAATTT-3´ | JF495479 |  |  | Mameaux *et al*., 2012 |
| HvCKX2.1 | 5´-TCCCCGCTGGCTTGATC-3´ | JF495488 |  | OsCKX2/ZmCKX5 | Mameaux *et al*., 2012 |
|  | 5´-CATGCATATCGCATCAGATAGCT-3´ |  |  |  |  |
| HvCKX2.2 | 5´-CCCGTGGTCAGAGAATTTTCA-3´ | AK373850 |  | OsCKX2/ZmCKX5 | Matsumoto *et al*., 2011 |
|  | 5´-GGGCCAATCGATTAACTTCGT-3´ | JF495489 |  |  | Mameaux *et al*., 2012 |
| HvCKX3 | 5´-GGGCCAGGCCAAGGTATATT-3´ | JF495480 |  | OsCKX3/ZmCKX6 | Mameaux *et al*., 2012 |
|  | 5´-ATGCTGTCAGCTTGCATAAACC-3´ |  |  |  |  |
| HvCKX4 | 5´-AGGCGGCCCGAAGAAC-3´ | BJ479455 | Contig24300_at | OsCKX4/ZmCKX2-3 | Mameaux *et al*., 2012 |
|  | 5´-TGCTGTACTCTGAAGTGTGTGTTGAG-3´ | BJ479606 |  |  |  |
|  |  | JF495481 |  |  |  |
| HvCKX5 | 5´-CCGCTCCTGTCCGATTCTT-3´ | AK370106 | HV_CEa0008F11r2_at | OsCKX5/ZmCKX4-4b | Matsumoto *et al.,* 2011 |
|  | 5´-GCTAGCTTCCTGAATGTCATGTTAAG-3´ | JF495482 |  |  | Mameaux *et al*., 2012 |
| HvCKX7 | 5´-TGACCCCCACGCCATAAT-3´ | JF495483 |  | OsCKX6-7/ZmCKX7-8 | Mameaux *et al*., 2012 |
|  | 5´-GGCACACTTGGCAATTGAAA-3´ |  |  |  |  |
| HvCKX8 | 5´-CTCGCGTAGAAGACATAGACTCGTA-3´ | JF495487 | Contig13202_at | OsCKX8/ZmCKX11-12 | Mameaux *et al*., 2012 |
|  | 5´-CGCGGACGTACTTGAACAGA-3´ | AJ234763 |  |  | Michálek *et al*., 1999 |
| HvCKX9 | 5´-TGGAGCAATATGTCTATGTTAGTATGGA-3´ | AF540382 | Contig25363_at | OsCKX9/- | Galuszka *et al*., 2004 |
|  | 5´-TCTTCACGCTGCAGTTCGTT-3´ | JF495484 |  |  | Mameaux *et al*., 2012 |
| HvCKX10 | 5´-ACGACGCCCGCTACAATC-3´ | JF495485 |  | OsCKX10/ZmCKX9 | Mameaux *et al*., 2012 |
|  | 5´-CGCGGACGTACTTGAACAGA-3´ |  |  |  |  |
| HvCKX11 | 5´-TCAAGACCTACTTCCCGCACTAC-3´ | AK355215 |  | OsCKX11/ZmCKX10 | Matsumoto *et al*., 2011 |
|  | 5´-GGAAGCGGGCCCAGTT-3´ | JF495486 |  |  | Mameaux *et al*., 2012 |
|  |  | CA031729 |  |  | Zhang *et al*., 2004 |
| ZmCKX1 | 5´- CGGTGTCGCTGCTCTTCTC-3´ | AF044603 |  | HvCKX1/OsCKX1 | Morris *et al*.,1999 |
|  | 5´- CGCCAGGTAGGTCTTGTACTGGAT-3´ | Y18377 |  |  | Houba-Hérin *et al*.,1999 1999 |
|  | 5´- TGGCGAGGCTGCAGGAGCAGA-3´ |  |  |  |  |
| HvPHT | 5´-AACTTTCTGGGCCTGCTCTTT-3´ | AF543197.1 |  |  | Rae *et al*., 2003 |
|  | 5´-AGAGCTCCTCGAGCGACTTG-3´ |  |  |  |  |
|  | 5´-CCCTGCTGGTGCCGGAGTCC-3´ |  |  |  |  |
| HvACT | 5´-TGTTGACCTCCAAAGGAAGCTATT-3´ | AK248432.1 | Contig11094_at |  | Sato *et al*., 2009 |
|  | 5´-TCAACAGCAGGTCTTGCACC-3´ |  |  |  |  |
|  | 5´-TGTAGTATTCAGCTGGTTGGTGGCACAGC-3´ |  |  |  |  |
| HvEF2 | 5´-AAGTCCTGCCGCACTGTCAT-3´ | AK250137.1 | Contig827_x_at |  | Sato *et al*., 2009 |
|  | 5´-GGGCGAGCTTCCATGTAAAG-3´ |  | Contig626_x_at |  |  |
|  | 5´-AGCAAGTCCCCCAACAAGCATAACCG-3´ |  |  |  |  |

The closest rice and maize orthologous genes together with GenBank accession numbers, reference and probe names on the Barley Affymetrix chip are listed.

Houba-Hérin N, Pethe C, d’Alayer J, Laloue M (1999) Cytokinin oxidase from Zea mays: purification, cDNA cloning and expression in moss protoplasts. Plant J 17: 615-626.

Matsumoto T, Tanaka T, Sakai H, Amano N, Kanamori H, *et al.* (2011) Comprehensive sequence analysis of 24,783 barley full-length cDNAs derived from 12 clone libraries. Plant Phys 156: 20-28.

Michalek W, Kunzel G, Graner A (1999) Sequence analysis and gene identification in a set of mapped markers in barely (*Hordeum vulgare*). Genome 42: 849-853.

Morris RO, Bilyeu KD, Laskey JG, Cheikh NN (1999) Isolation of a gene encoding a glycosylated cytokinin oxidase from maize. Biochem Biophys Res Comm 255: 328-333.

Rae AL, Cybinski DH, Jarmey JM, Smith FW (2003) Characterization of two phosphate transporters from barely; evidence for diverse function and kinetic properties among members of the PHT1 family. Plant Mol Biol 53: 27-36.

Sato K, Shin-I T, Seki M, Shinozaki K, Yoshida H, *et al.* (2009) Development of 5006 full-length cDNAs in barley: A tool for accessing cereal genomics resources. DNA Res 16: 81-89.

Tamura K, Peterson D, Peterson N, Stecher G, Nei M, Kumar S (2011) **MEGA5: Molecular Evolutionary Genetics Analysis using Maximum Likelihood, Evolutionary Distance, and Maximum Parsimony Methods.** Mol Biol Evol 28: 2731-2739.

Zhang H, Sreenivasulu N, Weschke W, Stein N, Rudd S, *et al*. (2004) Large-scale analysis of the barley transcriptome based on expressed sequence tags. Plant J 40: 276-290.
