# Supplementary material for: Overexpression of Cytokinin Dehydrogenase Genes in Barley (Hordeum vulgare cv. Golden Promise) Fundamentally Affects Morphology and Fertility
Source: PLoS One. 2013 Nov 15;8(11):e79029. doi: 10.1371/journal.pone.0079029 (PMC3829838; doi:10.1371/journal.pone.0079029)
Supplement: Table S2 — Sequences of primers and Taqman probes used for qPCR. Contig numbers containing ORFs of assigned putative genes with indicated numbers and positions (lengths) of exons, the closest rice and maize orthologous genes together with GenBank accession numbers or previously described ESTs are listed. (DOCX) [file pone.0079029.s006.docx]

**Table S2. Sequences of primers and Taqman probes used for qPCR.**

| Gene | Sequence of forward and reverse primers (and Taqman probe) | # of exons | Contig name^^ | Exon start | Exon stop | The closest ortholog | GenBank # EST |
| --- | --- | --- | --- | --- | --- | --- | --- |
| HvIPT1 | 5´-ACTTCGGGTGGCATTTGC -3´ | 10 | morex_contig_71263 L | 434 | 706 | OsIPT9/ZmIPT1 | CA009672.1 |
|  | 5´-CCACGAGTCACCAGTGGTACA-3´ |  |  | 823 | 900 |  | BU987274.1 |
|  |  |  |  | 999 | 1068 |  | GH225338.1 |
|  |  |  |  | 1207 | 1288 |  |  |
|  |  |  |  | 1390 | 1483 |  |  |
|  |  |  |  | 1586 | 1661 |  |  |
|  |  |  |  | 1810 | 2165 |  |  |
|  |  |  |  | 2259 | 2337 |  |  |
|  |  |  |  | 2428 | 2589 |  |  |
|  |  |  |  | 2760 | 2893 |  |  |
| HvIPT2 | 5´-GCGGCTCCAACAGGTACGT-3´ | 1 | morex_contig_140856 | 1369 | 2223 | OsIPT1-2/ZmIPT2 | BU997247.1 |
|  | 5´-CCGGCGGAGGACTTCTG-3´ |  |  |  |  |  | CB858532.1 |
| HvIPT3 | 5´-TACCTCGCTGGGCGTAAGA-3´ | 1 | morex_contig_37390 | 4119 | 5179 | OsIPT8/ZmIPT3 |  |
|  | 5´-GATCAAGGAGAACACGAGGGTG-3´ |  |  |  |  |  |  |
| HvIPT4 | 5´-CGAGGTGGATGAGGCTATGG-3´ | 1 | morex_contig_1567227 | 4095 | 5273 | OsIPT1-2/ZmIPT2 | AK375377.1 |
|  | 5´-TTGCTGCAGTCGGTGACG-3´ |  |  |  |  |  |  |
| HvIPT5 | 5´-CGCGCCGCACAGACTACT-3´ | 1 | morex_contig_49383 | 1934 | 2971 | OsIPT4/ZmIPT5-6 |  |
|  | 5´-CCGGTGGAGCGAAGATAGG-3´ |  |  |  |  |  |  |
|  | 5´-CCATCGGCGTCCCAGAGCTTGAC-3´ |  |  |  |  |  |  |
| HvIPT7 | 5´-CGACGAGATCAAGGAGAACACA-3´ | 1 | morex_contig_66093 | 1409 | 2449 | OsIPT3/ZmIPT7 |  |
|  | 5´-CCGACCTTGAGCGAAAGC-3´ |  |  |  |  |  |  |
| HvIPT10 | 5´-CCTTACCATACAGCTCATTCCAGAA-3´ | 1 | morex_contig_38949 | 2682 | 4031 | OsIPT10/ZmIPT10 | CB864042.1 |
|  | 5´-TGATGCAACCTGTGAAACGAA-3´ |  |  |  |  |  | CB860233.1 |
| HvCKX3 | 5´-GGGCCAGGCCAAGGTATATT-3´ | 4 | morex_contig_37471 | 967 | 1541 | OsCKX3/ZmCKX6 | JF495480 |
|  | 5´-ATGCTGTCAGCTTGCATAAACC-3´ |  |  | 1688 | 1816 |  |  |
|  |  |  |  | 1952 | 2212 |  |  |
|  |  |  |  | 2305 | 2567 |  |  |
|  |  | 1 | morex_contig_63132 | 267 | 607 |  |  |
| HvCKX8 | 5´-CTCGCGTAGAAGACATAGACTCGTA-3´ | 4 | morex_contig_37316 | 3242 | 3838 | OsCKX8/ZmCKX11-12 | AJ234763 |
|  | 5´-CCCTACAAGAAAGGACACCTACCA-3´ |  |  | 3988 | 4116 |  | JF495487 |
|  |  |  |  | 4211 | 4469 |  |  |
|  |  |  |  | 4585 | ∞ |  |  |
|  |  | 2 | morex_contig_2522512 | ∞ | 52 |  |  |
|  |  |  |  | 71 | 243 |  |  |
| HvCKX10 | 5´-ACGACGCCCGCTACAATC-3´ | 2 | morex_contig_81495 | 526 | 1127 | OsCKX10/ZmCKX9 | JF495485 |
|  | 5´-CGCGGACGTACTTGAACAGA-3´ |  |  | 1267 | 2269 |  |  |

Contig numbers containing ORFs of assigned putative genes with indicated numbers and positions (lengths) of exons, the closest rice and maize orthologous genes together with GenBank accession numbers or previously described ESTs are listed;  ^♦^contigs are generated from rough barley genome sequence (Mayer *et al*., 2012).
