# Supplementary material for: Overexpression of Cytokinin Dehydrogenase Genes in Barley (Hordeum vulgare cv. Golden Promise) Fundamentally Affects Morphology and Fertility
Source: PLoS One. 2013 Nov 15;8(11):e79029. doi: 10.1371/journal.pone.0079029 (PMC3829838; doi:10.1371/journal.pone.0079029)
Supplement: Table S3 — Transcript abundance in indicated barley tissues. Abundance is expressed as number of transcripts per ng of total RNA amplified by qPCR with respect to primer pair efficiency. RNA from two biological replicates was transcribed in two independent reactions, and PCR was performed in duplicate. Mean values ± standard deviations are shown. (DOCX) [file pone.0079029.s007.docx]

| Gene | Leaf | | | | | | | | | | |
| --- | --- | --- | --- | --- | --- | --- | --- | --- | --- | --- | --- |
|  | 3-d-old Leaf | 7-d-old Leaf | 14-d-old/1^st^ Leaf | 14-d-old/2^nd^ Leaf | 21-d-old Leaf | 3-m-old/1^st^ Leaf | 4-m-old/1^st^ Leaf | 5-m-old/1^st^ Leaf | 3-m-old/4^th^ Leaf | 4-m-old/4^th^ Leaf | 5-m-old/4^th^ Leaf |
| *HvCKX* genes | | | |  |  |  |  |  |  |  |  |
| *CKX1* | 4.2 ± 1.1 | 2.2 ± 0.5 | 1.8 ± 0.7 | < 1 | 2.8 ± 1.8 | 2.5 ± 0.5 | 5.2 ± 0.7 | 4.0 ± 1.2 | 14.6 ± 5.6 | 67.2 ± 28.6 | 492.2 ± 78.9 |
| *CKX2.1* | 1.7 ± 0.4 | < 1 | < 1 | 3.7 ± 1.7 | 11.2 ± 1.1 | 1.1 ± 0.2 | 1.5 ± 0.7 | 2.2 ± 1.2 | 2.8 ± 1.0 | < 1 | 3.9 ± 0.7 |
| *CKX2.2* | 166.6 ± 55.3 | 81.4 ± 12.9 | 43.1 ± 17.4 | 197.5 ± 125.5 | 1,838.7 ± 75.4 | 4,732.0 ± 2,429.3 | 74.0 ± 35.1 | 69.6 ± 38.7 | 72.5 ± 44.9 | 110.0 ± 33.3 | 398.7 ± 176.4 |
| *CKX3* | 4.0 ± 0.7 | 6.2 ± 3.2 | 181.7 ± 90.5 | 41.3 ± 21.9 | 71.3 ± 1.9 | 32.9 ± 3.5 | 6.4 ± 3.7 | 4.0 ± 1.7 | 21.7 ± 4.5 | 21.6 ± 10.5 | 12.3 ± 5.4 |
| *CKX4* | 11.0 ± 4.9 | 391.2 ± 3.7 | 360.6 ± 14.9 | 291.1 ± 3.0 | 300.1 ± 30.0 | 276.4 ± 72.9 | 636.7 ± 261.7 | 409.3 ± 22.4 | 293.7 ± 75.2 | 2,221.0 ± 1,590.4 | 2,163.5 ± 1,493.6 |
| *CKX5* | 283.5 ± 106.9 | 435.0 ± 57.9 | 260.8 ± 3.7 | 266.4 ± 69.6 | 346.9 ± 11.8 | 1,697.2 ± 606.3 | 41.7 ± 22.5 | 124.4 ± 66.1 | 287.3 ± 99.7 | 5.7 ± 2.1 | 13.0 ± 7.1 |
| *CKX7* | < 1 | < 1 | 6.5 ± 3.2 | < 1 | < 1 | < 1 | 4.8 ± 1.5 | 4.8 ± 1.5 | < 1 | < 1 | < 1 |
| *CKX8* | 94.1 ± 32.4 | 311.1 ± 53.7 | 1,542.6 ± 84.3 | 380.4 ± 54.8 | 985.5 ± 300.3 | 211.1 ± 48.6 | 436.3 ± 165.3 | 560.1 ± 227.0 | 535.6 ± 229.0 | 2,273.0 ± 1,221.7 | 1,424.4 ± 500.7 |
| *CKX9* | 2.6 ± 0.5 | 16.7 ± 9.5 | 76.6 ± 5.7 | 6.8 ± 1.6 | 1.0 ± 0.4 | 14.5 ± 0.7 | 127.1 ± 8.0 | 244.4 ± 69.3 | 8.1 ± 3.7 | 278.3 ± 89.5 | 161.7 ± 87.4 |
| *CKX10* | 21.4 ± 2.8 | 13.9 ± 7.0 | 22.9 ± 5.7 | 22.7 ± 12.8 | 105.5 ± 14.0 | 5.2 ± 0.6 | 282.7 ± 13.3 | 5.6 ± 2.5 | 48.5 ± 17.8 | 37.4 ± 3.4 | 7.0 ± 0.8 |
| *CKX11* | 176.9 ± 80.5 | 1,008.5 ± 115.6 | 1,236.5 ± 164.1 | 853.8 ± 123.4 | 355.9 ± 72.0 | 468.8 ± 111.6 | 1,499.5 ± 916.7 | 1,649.7 ± 571.6 | 639.0 ± 289.2 | 339.8 ± 40.5 | 465.7 ± 203.3 |
| *HvIPT* genes | | | | | | | |  |  |  |  |
| *IPT1* | 10.8 ± 2.5 | 72.9 ± 34.9 | 42.8 ± 5.3 | 68.0 ± 15.6 | 105.2 ± 21.2 | 23.6 ± 12.4 | 49.3 ± 27.4 | 24.4 ± 2.8 | 20.6 ± 15.7 | 103.0 ± 14.0 | 40.3 ± 24.4 |
| *IPT2* | 63.2 ± 30.9 | 70.1 ± 11.0 | 5.5 ± 3.3 | 20.6 ± 14.9 | 421.8 ± 124.2 | 55.7 ± 10.5 | 98.6 ± 42.0 | 27.9 ± 5.7 | 590.3 ± 216.3 | 431.3 ± 248.8 | 6.6 ± 4.0 |
| *IPT3* | 4.1 ± 1.5 | 33.0 ± 15.3 | 3.2 ± 1.1 | 11.2 ± 1.6 | 23.1 ± 0.7 | 19.9 ± 2.8 | 18.4 ± 3.0 | 3.8 ± 0.4 | 54.5 ± 24.3 | 7.8 ± 3.4 | 11.4 ± 3.0 |
| *IPT4* | 1.1 ± 0.9 | 2.6 ± 1.4 | 1.3 ± 0.1 | 2.4 ± 1.8 | < 1 | 15.5 ± 5.0 | 27.9 ± 11.2 | 1.1 ± 0.3 | 1.7 ± 0.7 | < 1 | 2.0 ± 0.8 |
| *IPT5* | 235.2 ± 26.3 | 292.9 ± 47.5 | 808.1 ± 328.1 | 448.1 ± 221.8 | 859.1 ± 180.3 | 1,517.6 ± 59.9 | 1,338.7 ± 33.8 | 2,821.1 ± 174.1 | 3,098.1 ± 1,236.9 | 2,498.8 ± 795.7 | 4,022.5 ± 595.3 |
| *IPT7* | 3.2 ± 1.3 | 1.2 ± 0.9 | 11.9 ± 5.4 | 17.3 ± 9.4 | 2.4 ± 0.1 | 10.8 ± 6.3 | 3.4 ± 0.8 | 32.2 ± 11.1 | 91.6 ± 33.9 | 9.8 ± 4.4 | 10.1 ± 3.4 |
| *IPT10* | 26.5 ± 9.0 | 34.4 ± 18.4 | 8.8 ± 0.4 | 124.0 ± 50.1 | 227.1 ± 47.9 | 5.1 ± 2.1 | 13.0 ± 7.7 | 18.4 ± 6.4 | 11.8 ± 2.4 | 6.4 ± 2.6 | 11.7 ± 5.5 |

**Table S3.** **Transcript abundance in indicated barley tissues.** Abundance is expressed as number of transcripts per ng of total RNA ampliﬁed by qPCR with respect to primer pair efﬁciency. RNA from at least two biological replicates was transcribed in two independent reactions, and PCR was performed in duplicate. Mean values ± standard deviations are shown.

| Gene | Flowers (before pollination) | Husk | Spikes | Embryo | Endosperm | Stem | | | | | |
| --- | --- | --- | --- | --- | --- | --- | --- | --- | --- | --- | --- |
|  |  |  |  |  |  | 7-d-old Coleoptile | 14-d-old Coleoptile | 3-m-old stem base | Node | 3-m-old Stem | 4 + 5-m-old Stem |
| *HvCKX* genes | | | |  |  |  |  |  |  |  |  |
| *CKX1* | 698.7 ± 332.3 | 243.8 ± 64.6 | 152.4 ± 108.8 | 10,286.9 ± 4,955.2 | 265.6 ± 119.8 | 18.0 ± 11.8 | 7.8 ± 3.3 | < 1 | 5.1 ± 2.9 | < 1 | 3.0 ± 1.3 |
| *CKX2.1* | 5.9 ± 1.2 | 536.5 ± 269.3 | 2.9 ± 1.7 | 75.2 ± 18.5 | 4.8 ± 2.1 | < 1 | 1.4 ± 1.0 | 3.9 ± 1.4 | 2.8 ± 0.9 | 1.9 ± 0.4 | 2.8 ± 1.0 |
| *CKX2.2* | 281.6 ± 13.5 | 967.8 ± 447.3 | 2,284.2 ± 1,098.0 | 124.5 ± 33.9 | 19.7 ± 8.5 | 14.6 ± 4.1 | 11.2 ± 6.2 | 258.0 ± 66.8 | 78.1 ± 25.1 | 11.3 ± 2.3 | 10.2 ± 4.3 |
| *CKX3* | 4.3 ± 1.2 | 29.1 ± 14.0 | 11.4 ± 2.6 | 31.2 ± 17.1 | 34.8 ± 21.6 | 267.0 ± 40.2 | 45.3 ± 21.3 | 247.5 ± 50.4 | 133.2 ± 24.5 | 18.7 ± 4.2 | 45.2 ± 11.6 |
| *CKX4* | 398.5 ± 70.5 | 301.8 ± 61.4 | 327.3 ± 65.0 | 611.6 ± 21.0 | 4.2 ± 1.1 | 170.6 ± 20.3 | 194.4 ± 55.5 | 224.2 ± 18.5 | 595.1 ± 55.8 | 309.1 ± 4.9 | 275.5 ± 45.1 |
| *CKX5* | 107.7 ± 43.6 | 76.5 ± 37.7 | 134.4 ± 76.1 | 75.6 ± 15.0 | 8.4 ± 1.4 | 286.6 ± 14.7 | 402.6 ± 188.4 | 331.2 ± 83.2 | 364.5 ± 72.0 | 104.3 ± 4.1 | 13.9 ± 3.9 |
| *CKX7* | < 1 | < 1 | < 1 | 2.5 ± 1.5 | 5.0 ± 1.6 | 2.5 ± 0.2 | 2.1 ± 0.9 | 17.5 ± 3.9 | 2.9 ± 0.2 | < 1 | < 1 |
| *CKX8* | 737.7 ± 397.7 | 99.8 ± 8.0 | 326.1 ± 143.1 | 431.7 ± 148.9 | 53.1 ± 24.0 | 80.5 ± 26.1 | 227.7 ± 94.0 | 126.3 ± 38.7 | 79.2 ± 33.5 | 72.1 ± 4.5 | 170.8 ± 76.1 |
| *CKX9* | 11.6 ± 5.2 | 2.3 ± 0.7 | 27.5 ± 14.0 | 2.6 ± 0.8 | 1.7 ± 1.0 | 1.3 ± 0.8 | 23.0 ± 5.2 | 3.0 ± 1.2 | 20.2 ± 11.7 | < 1 | 20.0 ± 7.5 |
| *CKX10* | 2.6 ± 1.5 | 46.2 ± 31.8 | 29.3 ± 14.7 | 15.9 ± 3.9 | 34.3 ± 3.2 | 25.9 ± 5.5 | 13.7 ± 2.2 | 9.7 ± 2.1 | 20.2 ± 7.5 | 17.6 ± 2.3 | 3.4 ± 1.7 |
| *CKX11* | 2118.7 ± 406.6 | 93.2 ± 31.7 | 403.9 ± 186.0 | 633.4 ± 374.7 | 165.7 ± 84.0 | 753.5 ± 40.9 | 773.7 ± 181.4 | 256.3 ± 56.6 | 328.5 ± 130.3 | 95.1 ± 5.7 | 139.6 ± 16.8 |
| *HvIPT* genes | | | | | | | |  |  |  |  |
| *IPT1* | 16.6 ± 2.2 | 50.5 ± 21.6 | 37.4 ± 11.4 | 904.3 ± 191.4 | 22.4 ± 19.3 | 88.9 ± 6.9 | 50.6 ± 23.2 | 95.6 ± 38.2 | 64.4 ± 10.0 | 29.3 ± 8.5 | 107.4 ± 24.9 |
| *IPT2* | 3.0 ± 1.2 | 134.7 ± 20.0 | 435.4 ± 231.2 | 59.2 ± 24.8 | 212.6 ± 162.5 | 170.6 ± 0.0 | 646.1 ± 243.3 | 3.7 ± 0.9 | 9.1 ± 4.1 | 43.7 ± 22.3 | 9.7 ± 4.2 |
| *IPT3* | 4.3 ± 2.0 | 5.6 ± 1.2 | 23.6 ± 8.4 | 16.9 ± 9.0 | 13.0 ± 7.5 | 1.7 ± 0.1 | 3.1 ± 1.3 | 3.6 ± 1.8 | 34.3 ± 5.7 | 32.5 ± 9.7 | 22.9 ± 5.0 |
| *IPT4* | 1.3 ± 0.8 | 2.0 ± 0.9 | 2.1 ± 1.6 | 6.0 ± 4.6 | 1.1 ± 0.7 | 22.5 ± 8.2 | 84.5 ± 34.9 | 1.0 ± 0.6 | 168.3 ± 4.3 | 1.8 ± 0.8 | 1.5 ± 1.2 |
| *IPT5* | 147.9 ± 25.1 | 387.2 ± 144.0 | 5,374.5 ± 946.8 | n.d. | 726.7 ± 6.0 | 1,400.2 ± 285.4 | 99.1 ± 44.1 | 196.2 ± 13.8 | 9,544.2 ± 2,146.5 | 1,148.4 ± 351.1 | 8,314.4 ± 1,015.7 |
| *IPT7* | < 1 | 2.3 ± 1.5 | 107.1 ± 31.6 | 1.4 ± 0.6 | 3.4 ± 2.9 | 4.9 ± 0.4 | 1.9 ± 0.9 | 4.9 ± 1.4 | 4.1 ± 2.5 | 64.8 ± 10.9 | 7.1 ± 2.1 |
| *IPT10* | 8.5 ± 0.2 | 10.4 ± 4.8 | 34.3 ± 14.3 | 91.2 ± 35.0 | 2.2± 1.4 | 4.5 ± 1.0 | 4.7 ± 2.0 | 116.1 ± 17.3 | 10.2 ± 1.6 | 7.3 ± 0.6 | 23.3 ± 3.3 |

| Gene | Root | | | | | | | | | | |
| --- | --- | --- | --- | --- | --- | --- | --- | --- | --- | --- | --- |
|  | 3-d-old Root | 5-d-old Root | 7-d-old Root | 14-d-old Root | 21-d-old Root | 3-m-old Root | 4-m-old Root | 5-m-old Root | 5-d-old Root apex | | 21-d-old Hairy root |
| *HvCKX* genes | | | |  |  |  |  |  |  |  | |
| *CKX1* | 45.0 ± 20.7 | 96.1 ± 19.5 | 45.8 ± 29.4 | 124.0 ± 14.1 | 815.1 ± 54.7 | 232.0 ± 66.6 | 3.9 ± 1.9 | 242.7 ± 68.4 | 176.6 ± 7.7 | | 1,703.6 ± 78.7 |
| *CKX2.1* | < 1 | < 1 | 114.3 ± 9.8 | 37.2 ± 22.8 | 10.7 ± 1.5 | 2.2 ± 1.1 | 10.2 ± 5.0 | 3.3 ± 1.4 | 2.1 ± 1.5 | | 20.9 ± 11.4 |
| *CKX2.2* | 75.6 ± 29.9 | 32.5 ± 2.5 | 23.7 ± 9.7 | 19.0 ± 7.2 | 4.0 ± 1.1 | 20.3 ± 3.2 | 7.7 ± 2.6 | 37.2 ± 0.8 | 42.0 ± 10.6 | | 241.9 ± 92.8 |
| *CKX3* | 269.3 ± 86.6 | 275.5 ±16.3 | 319.7 ± 13.8 | 598.9 ± 81.9 | 1,226.0 ± 157.6 | 194.0 ± 21.7 | 31.0 ± 12.3 | 76.1 ± 30.6 | 552.3 ± 238.1 | | 1,617.5 ± 340.7 |
| *CKX4* | 7.9 ± 0.1 | 15.0 ± 0.7 | 22.8 ± 7.2 | 4.5 ± 1.1 | 127.7 ± 64.3 | 338.0 ± 120.3 | 355.1 ± 38.5 | 1,832.7 ± 362.7 | 16.5 ± 7.1 | | 81.4 ± 35.5 |
| *CKX5* | 4.8 ± 1.4 | 8.7 ± 1.2 | 4.6 ± 2.6 | 6.8 ± 1.9 | 65.2 ± 14.6 | 244.5 ± 4.6 | 42.5 ± 2.7 | 227.2 ± 15.7 | 81.9 ± 23.8 | | 53.4 ± 6.8 |
| *CKX7* | 48.8 ± 25.3 | 36.5 ± 4.6 | 84.8 ± 23.7 | 16.0 ± 0.6 | 25.5 ± 1.7 | 5.5 ± 1.1 | 3.3 ± 1.6 | 2.7 ± 1.5 | 31.5 ± 11.0 | | 10.1 ± 2.6 |
| *CKX8* | 204.6 ± 77.3 | 129.3 ± 3.3 | 130.7 ± 7.0 | 314.1 ± 33.2 | 331.7 ± 5.3 | 87.9 ± 25.1 | 19.5 ± 9.0 | 48.9 ± 23.3 | 195.8 ± 16.9 | | 231.6 ± 50.0 |
| *CKX9* | 33.2 ± 17.4 | 5.1 ± 0.6 | 13.1 ± 6.4 | 6.1 ± 2.8 | 4.0 ± 0.4 | 2.5 ± 1.0 | 2.4 ± 1.3 | 15.7 ± 1.3 | 7.9 ± 4.7 | | 5.2 ± 0.1 |
| *CKX10* | 7.1 ± 1.2 | 7.9 ± 0.9 | 5.3 ± 0.3 | 6.0 ± 3.2 | 5.6 ± 1.5 | 5.3 ± 1.9 | 2.8 ± 0.8 | 1.8 ± 0.6 | 9.3 ± 1.4 | | 9.7 ± 5.1 |
| *CKX11* | 854.4 ± 213.1 | 506.2 ± 304.8 | 455.1 ± 28.5 | 302.9 ± 88.2 | 506.7 ± 69.7 | 340.6 ± 41.2 | 127.1 ± 13.4 | 163.9 ± 49.6 | 530.3 ± 89.7 | | 602.8 ± 31.1 |
| *HvIPT* genes | | | | | | | |  |  | |  |
| *IPT1* | 74.6 ± 8.9 | 89.6 ± 1.4 | 60.7 ± 16.2 | 82.2 ± 26.6 | 183.1 ± 10.1 | 37.7 ± 3.8 | 23.0 ± 12.0 | 33.8 ± 12.9 | 303.7 ± 44.6 | | 264.3 ± 20.3 |
| *IPT2* | 72.6 ± 15.7 | 58.3 ± 25.1 | 64.6 ± 15.9 | 28.9 ± 18.0 | 182.4 ± 21.8 | 60.6 ± 7.0 | 1.8 ± 0.0 | 25.3 ± 10.8 | 64.6 ± 10.4 | | 210.0 ± 3.3 |
| *IPT3* | 8.3 ± 2.0 | 6.7 ± 2.4 | 4.5 ± 0.5 | 6.4 ± 2.6 | 12.1 ± 2.9 | 12.1 ± 4.2 | 4.2 ± 0.5 | 5.3 ± 0.0 | 26.7 ± 4.9 | | 38.3 ± 13.8 |
| *IPT4* | 3.9 ± 2.7 | 34.3 ± 0.6 | 6.3 ± 3.2 | 5.7 ± 3.6 | 31.0 ± 3.4 | 31.4 ± 11.7 | 33.5 ± 2.8 | 19.4 ± 2.0 | 3.0 ± 0.8 | | 38.2 ± 12.2 |
| *IPT5* | 1,863.5 ± 275.5 | 1,561.8 ± 757.8 | 326.2 ± 192.4 | 1,646.5 ± 792.4 | 1,491.1 ± 24.8 | 450.4 ± 65.4 | 971.9 ± 558.0 | 432.8 ± 115.2 | 1,468.0 ± 62.6 | | 1,745.9 ± 173.6 |
| *IPT7* | < 1 | n.d. | 1.6 ± 0.0 | < 1 | 6.4 ± 0.8 | < 1 | 3.5 ± 1.9 | 1.8 ± 1.4 | 1.8 ± 0.6 | | 10.5 ± 4.1 |
| *IPT10* | 16.5 ± 1.5 | 21.2 ± 2.1 | 5.8 ± 1.0 | 14.1 ± 0.4 | 27.9 ± 6.0 | 18.6 ± 2.6 | 4.2 ± 2.6 | 9.2 ± 4.3 | 144.6 ± 16.0 | | 36.9 ± 4.3 |

| Gene | Root | | |
| --- | --- | --- | --- |
|  | 3-m-old Adventive root | 4-m-old Adventive root | 5-m-old Adventive root |
| *HvCKX* genes | | | |
| *CKX1* | 247.8 ± 113.3 | 8.1 ± 2.0 | 53.1 ± 32.7 |
| *CKX2.1* | 1.8 ± 0.1 | 1.6 ± 0.4 | 2.5 ± 1.5 |
| *CKX2.2* | 94.2 ± 50.5 | 19.6 ± 6.6 | 65.1 ± 12.4 |
| *CKX3* | 312.9 ± 134.2 | 21.6 ± 13.4 | 15.8 ± 2.5 |
| *CKX4* | 825.3 ± 347.1 | 245.9 ± 110.5 | 442.9 ± 255.6 |
| *CKX5* | 808.2 ± 211.7 | 49.2 ± 22.6 | 83.6 ± 30.9 |
| *CKX7* | 36.7 ± 5.1 | < 1 | 8.3 ± 0.3 |
| *CKX8* | 238.8 ± 54.4 | 29.2 ± 10.6 | 8.3 ± 1.8 |
| *CKX9* | 11.7 ± 0.9 | 2.4 ± 1.3 | 1.8 ± 0.9 |
| *CKX10* | 5.4 ± 0.3 | 1.9 ± 1.3 | 3.3 ± 0.1 |
| *CKX11* | 487.2 ± 58.4 | 112.9 ± 19.3 | 67.3 ± 4.8 |
| *HvIPT* genes | |  |  |
| *IPT1* | 66.8 ± 0.3 | 16.3 ± 5.1 | 7.8 ± 0.8 |
| *IPT2* | 18.5 ± 9.4 | 3.6 ± 0.1 | 39.3 ± 5.7 |
| *IPT3* | 10.4 ± 6.0 | 10.2 ± 1.4 | 9.5 ± 2.1 |
| *IPT4* | 22.3 ± 12.6 | 21.5 ± 0.7 | 16.4 ± 3.7 |
| *IPT5* | 850.1 ± 9.0 | 1,272.0 ± 49.2 | 965.7 ± 103.1 |
| *IPT7* | 3.9 ± 0.1 | 3.9 ± 1.4 | 2.4 ± 0.5 |
| *IPT10* | 31.8 ± 1.5 | 4.5 ± 1.9 | 3.7 ± 1.7 |
