# Supplementary material for: Overexpression of Cytokinin Dehydrogenase Genes in Barley (Hordeum vulgare cv. Golden Promise) Fundamentally Affects Morphology and Fertility
Source: PLoS One. 2013 Nov 15;8(11):e79029. doi: 10.1371/journal.pone.0079029 (PMC3829838; doi:10.1371/journal.pone.0079029)
Supplement: Table S4 — Relative substrate specificity of ZmCKX1 and HvCKX9. Activity was measured as described in the Materials and Methods section (100 mM McIlvaine buffer, pH 6.0, with 0.5 mM 2,3-dimetoxy-5-methyl-1,4-benzoquinone as an electron acceptor and 0.25 mM substrate) with recombinant ZmCKX1 prepared in Pichia pastoris [21] and extract from tobacco constitutively overexpressing HvCKX9 [11]. Specific activity with iP considered as 100% was 752 nkat mg−1 for ZmCKX1 and 4.2 pkat mg−1 for HvCKX9. (DOCX) [file pone.0079029.s008.docx]

**Table S4. Relative substrate specificity of ZmCKX1 and HvCKX9.**

| Substrate | Relative specific activity (%) | |
| --- | --- | --- |
|  | ZmCKX1 | HvCKX9 |
| *N*^6^-isopentenyladenine | 100 | 100 |
| *N*^6^-isopentenyladenine-9-riboside | 65 | 410 |
| *N*^6^-isopentenyladenine-9-glucoside | 2 | 760 |
| *N^6^*-isopentenyladenine-9-riboside-5´-monophosphate | 5 | 490 |
| *Trans*-zeatin | 120 | 550 |
| *Trans*-zeatin-9-riboside | 20 | 140 |
| *Cis*-zeatin | 10 | n.d. |
| Dihydrozeatin | 0 | 0 |

Activity was measured as described in the *Materials and Methods* section (100 mM McIlvaine buffer, pH 6.0, with 0.5 mM 2,3-dimetoxy-5-methyl-1,4-benzoquinone as an electron acceptor and 0.25 mM substrate) with recombinant ZmCKX1 prepared in *Pichia pastoris* [21] and extract from tobacco constitutively overexpressing HvCKX9 [11]; specific activity with iP considered as 100% was 752 nkat mg^-1^ for ZmCKX1 and 4.2 pkat mg^-1^ for HvCKX9; n.d. – not determined
