# Supplementary material for: Overexpression of Cytokinin Dehydrogenase Genes in Barley (Hordeum vulgare cv. Golden Promise) Fundamentally Affects Morphology and Fertility
Source: PLoS One. 2013 Nov 15;8(11):e79029. doi: 10.1371/journal.pone.0079029 (PMC3829838; doi:10.1371/journal.pone.0079029)
Supplement: Table S5 — Endogenous CK levels (pmol g−1 FW) in leaves and roots of T0 barley plants transformed with Ubi::ZmCKX1 and PHT::ZmCKX1 . The analyzed CKs included: free bases of trans-zeatin (tZ), cis-zeatin (cZ), dihydrozeatin (DHZ) and isopentenyladenine (iP); their N9-ribosides (tZR, cZR, DHZR, iPR); O-glucosides (tZOG, cZOG, DHZOG); O-glucoside-N9-ribosides (tZROG, cZROG, DHZROG); N9-glucosides (tZ9G, cZ9G, DHZ9G, iP9G); and N9-riboside-5′-monophosphates (tZR5′MP, cZR5′MP, DHZ5′MP, iPR5′MP). Sum of free bases and N9-ribosides are considered to comprise the pool of active CKs. Mean values ± standard deviations from two samples derived from three pooled leaves and cut roots from the bottom of the root system are presented. (DOCX) [file pone.0079029.s009.docx]

**Table S5. Endogenous CK levels (pmol g^-1^ FW) in leaves and roots of T0 barley plants transformed with *Ubi::ZmCKX1* and *PHT::ZmCKX1*.**

| CK | CTRL | Ubi::ZmCKX1 | CTRL | Pht::ZmCKX1 | CK | CTRL | Ubi::ZmCKX1 | CTRL | Pht:ZmCKX1 |
| --- | --- | --- | --- | --- | --- | --- | --- | --- | --- |
| Roots | | | | | | | | | |
| tZ | 1.12 ± 0.51 | 0.39 ± 0.09 | 0.21 ± 0.06 | 0.39 ± 0.11 | cZ | 1.40 ± 0.32 | 4.55 ± 1.32 | 1.61 ± 0.34 | 0.42 ± 0.12 |
| tZR | 0.15 ± 0.02 | 0.99 ± 0.27 | 0.70 ± 0.34 | 0.39 ± 0.14 | cZR | 3.61 ± 0.76 | 5.73 ± 1.63 | 3.62 ± 1.39 | 3.54 ± 1.52 |
| tZOG | 0.57 ± 0.12 | 1.26 ± 0.43 | 0.99 ± 0.28 | 0.87 ± 0.23 | cZOG | 8.10 ± 2.45 | 12.12 ± 3.67 | 13.42 ± 4.78 | 6.62 ± 2.19 |
| tZROG | 0.11 ± 0.03 | 0.15 ± 0.05 | 0.21 ± 0.08 | 0.24 ± 0.06 | cZROG | 8.19 ± 3.21 | 10.45 ± 3.71 | 7.23 ± 2.60 | 7.86 ± 2.68 |
| tZ9G | 22.58 ± 3.87 | 82.31 ± 9.56 | 45.95 ± 6.76 | 22.28 ± 4.45 | cZ9G | 0.81 ± 0.21 | 0.66 ± 0.25 | 2.23 ± 0.78 | 0.50 ± 0.23 |
| tZR5´MP | 0.37 ± 0.16 | 0.48 ± 0.13 | u.d.l. | u.d.l. | cZR5´MP | 2.72 ± 0.87 | 5.36 ± 2.63 | 14.75 ± 3.85 | 7.09 ± 2.04 |
| **Active** | **1.27** | **1.38** | **0.91** | **0.77** | **Active** | **5.02** | **10.28** | **5.23** | **3.97** |
| **Total** | **24.90** | **85.57** | **48.06** | **24.16** | **Total** | **24.83** | **38.88** | **42.85** | **26.04** |
| DHZ | 0.09 ± 0.02 | 0.23 ± 0.07 | u.d.l. | u.d.l. | iP | 0.19 ± 0.02 | 0.53 ± 0.02 | 0.97 ± 0.02 | 0.14 ± 0.02 |
| DHZR | 0.13 ± 0.05 | 0.47 ± 0.15 | u.d.l. | u.d.l. | iPR | 2.65 ± 0.05 | 3.95 ± 0.05 | 3.20 ± 0.05 | 3.32 ± 0.05 |
| DHZOG | 0.39 ± 0.12 | 1.71 ± 0.62 | 0.83 ± 0.31 | 0.41 ± 0.17 | iP9G | 7.89 ± 0.12 | 17.28 ± 0.12 | 15.03 ± 0.12 | 16.81 ± 0.12 |
| DHZROG | 0.93 ± 0.33 | 2.63 ± 0.63 | 1.01 ± 0.29 | 1.06 ± 0.48 | iPR5´MP | 1.94 ± 0.33 | 1.77 ± 0.33 | 2.27 ± 0.33 | 4.51 ± 0.33 |
| DHZ9G | 0.10 ± 0.03 | 0.33 ± 0.13 | u.d.l. | u.d.l. |  |  |  |  |  |
| DHZ5´MP | 0.17 ± 0.06 | 0.73 ± 0.31 | u.d.l. | u.d.l. | **Active** | **2.84** | **4.48** | **4.17** | **3.46** |
| **Total** | **1.82** | **6.10** | **1.84** | **1.47** | **Total** | **12.66** | **23.53** | **21.47** | **24.78** |
| Leaves | | | | | | | | | |
| tZ | 0.28 ± 0.07 | 0.26 ± 0.09 | 0.13 ± 0.05 | 0.06 ± 0.02 | cZ | 0.69 ± 0.27 | 1.39 ± 0.65 | 0.20 ± 0.04 | 0.22 ± 0.10 |
| tZR | 0.10 ± 0.03 | 0.05 ± 0.27 | 0.01 ± 0.00 | 0.01 ± 0.00 | cZR | 6.58 ± 2.46 | 6.04 ± 2.04 | 1.89 ± 0.19 | 2.17 ± 0.82 |
| tZOG | 2.22 ± 0.92 | 1.30 ± 0.43 | 2.82 ± 0.79 | 1.04 ± 0.40 | cZOG | 20.96 ± 7.00 | 17.28 ± 5.29 | 30.11 ± 6.17 | 17.42 ± 4.16 |
| tZROG | 0.04 ± 0.01 | 0.02 ± 0.00 | 0.05 ± 0.02 | 0.02 ± 0.00 | cZROG | 1.55 ± 0.51 | 1.86 ± 0.61 | 4.63 ± 1.04 | 2.50 ± 1.01 |
| tZ9G | 9.39 ± 2.27 | 24.37 ± 5.96 | 5.21 ± 6.76 | 2.37 ± 0.45 | cZ9G | 0.10 ± 0.03 | 0.13 ± 0.05 | 0.05 ± 0.01 | 0.03 ± 0.00 |
| tZR5´MP | 1.27 ± 0.66 | 0.78 ± 0.43 | u.d.l. | u.d.l. | cZR5´MP | 14.83 ± 6.80 | 5.00 ± 2.35 | 12.87 ± 3.91 | 9.26 ± 4.07 |
| **Active** | **0.38** | **0.31** | **0.14** | **0.06** | **Active** | 7.27 | 7.43 | 2.09 | 2.39 |
| **Total** | **13.30** | **26.78** | **8.21** | **3.49** | **Total** | **44.71** | **31.70** | **49.76** | **31.60** |
| DHZ | 0.01 ± 0.00 | 0.01 ± 0.00 | u.d.l. | u.d.l. | iP | 1.56 ± 0.52 | 1.15 ± 0.42 | 0.12 ± 0.04 | 0.36 ± 0.12 |
| DHZR | 0.10 ± 0.03 | 0.07 ± 0.02 | u.d.l. | u.d.l. | iPR | 4.94 ± 1.32 | 3.50 ± 1.05 | 2.25 ± 0.75 | 3.44 ± 1.27 |
| DHZOG | 1.00 ± 0.42 | 1.70 ± 0.46 | 0.44 ± 0.17 | 0.24 ± 0.09 | iP9G | 2.01 ± 0.88 | 1.41 ± 0.74 | 1.51 ± 0.62 | 1.22 ± 0.51 |
| DHZROG | 0.10 ± 0.03 | 0.12 ± 0.04 | 0.11 ± 0.03 | 0.06 ± 0.03 | iPR5´MP | 3.00 ± 1.09 | 5.04 ± 1.53 | 1.16 ± 0.40 | 1.30 ± 0.43 |
| DHZ9G | 0.02 ± 0.00 | 0.07 ± 0.02 | u.d.l. | u.d.l. |  |  |  |  |  |
| DHZ5´MP | 1.27 ± 0.66 | 0.80 ± 0.26 | u.d.l. | u.d.l. | **Active** | **6.50** | **4.64** | **2.36** | **3.80** |
| **Total** | **2.50** | **2.76** | **0.55** | **0.30** | **Total** | **11.51** | **11.09** | **5.03** | **6.32** |

The analyzed CKs included: free bases of *trans*-zeatin (tZ), *cis*-zeatin (cZ), dihydrozeatin (DHZ) and isopentenyladenine (iP); their N^9^-ribosides (tZR, cZR, DHZR, iPR); O-glucosides (tZOG, cZOG, DHZOG); O-glucoside-N^9^-ribosides (tZROG, cZROG, DHZROG); N^9^-glucosides (tZ9G, cZ9G, DHZ9G, iP9G); and N^9^-riboside-5´-monophosphates (tZR5´MP, cZR5´MP, DHZ5´MP, iPR5´MP). Sum of free bases and N^9^-ribosides are considered to comprise the pool of active CKs. Mean values ± standard deviations from two samples derived from three pooled leaves and cut roots from the bottom of the root system are presented.
