# Supplementary material for: Overexpression of Cytokinin Dehydrogenase Genes in Barley (Hordeum vulgare cv. Golden Promise) Fundamentally Affects Morphology and Fertility
Source: PLoS One. 2013 Nov 15;8(11):e79029. doi: 10.1371/journal.pone.0079029 (PMC3829838; doi:10.1371/journal.pone.0079029)
Supplement: Table S6 — Transcript abundance of barley CKX and IPT gene families in T0-generation PHT::ZmCKX1 and control plants regenerated in vitro . Abundance is expressed as number of transcripts per ng of total RNA amplified by qPCR with respect to primer pair efficiency. RNA from two biological replicates was transcribed in two independent reactions, and PCR was performed in duplicate. Mean values ± standard deviations are shown. (DOCX) [file pone.0079029.s010.docx]

**Table S6. Transcript abundance of barley *CKX* and *IPT* gene families in T0-generation *PHT::ZmCKX1* and control plants regenerated *in vitro*.**

|  | 1–m-old Leaf | 2–m-old Leaf | 3–m-old Leaf | 4–m-old Leaf | 5-m-old Leaf |  | 1–m-old Leaf | 2–m-old Leaf | 3–m-old Leaf | 4–m-old Leaf | 5-m-old Leaf |
| --- | --- | --- | --- | --- | --- | --- | --- | --- | --- | --- | --- |
| ***PHT::ZmCKX1-1 (T0 generation)*** | | | | | | | | | | | |
| ***CKX1*** | 4.7 ± 0.2 | 2.2 ± 0.7 | < 1 | < 1 | < 1 | ***IPT1*** | 77.4 ± 11.8 | 144.9 ± 32.9 | 136.4 ± 25.7 | 334.3 ± 52.8 | 62.1 ± 18.9 |
| ***CKX2.2*** | 2,271.1 ± 1,059.1 | 429.3 ± 124.3 | 160.9 ± 6.5 | 410.5 ± 157.4 | 562.9 ± 191.7 | ***IPT3*** | 9.4 ± 2.5 | 22.6 ± 11.8 | 83.4 ± 35.8 | 24.8 ± 5.7 | 17.9 ± 7.8 |
| ***CKX4*** | 437.9 ± 97.5 | 203.2 ± 61.5 | 372.9 ± 168.4 | 358.1 ± 6.4 | 307.7 ± 51.4 | ***IPT5*** | 452.8 ± 285.2 | 680.4 ± 228.6 | 546.0 ± 123.1 | 1,059.7 ± 221.3 | 2,097.3 ± 273.3 |
| ***CKX5*** | 2,382.0 ± 224.7 | 61.9 ± 20.7 | 29.0 ± 12.3 | 159.4 ± 96.2 | 216.8 ± 93.2 | ***IPT7*** | 20.5 ± 9.8 | 17.8 ± 1.0 | 19.8 ± 6.4 | 25.2 ± 3.2 | 1.6 ± 0.7 |
| ***CKX7*** | 34.8 ± 7.6 | 17.0 ± 2.8 | 30.5 ± 8.8 | 51.2 ± 9.9 | 70.2 ± 23.8 | ***IPT10*** | 48.6 ± 23.9 | 82.0 ± 24.0 | 81.9 ± 14.4 | 15.3 ± 3.6 | 28.7 ± 10.3 |
| ***CKX8*** | 8,148.6 ± 1,582.3 | 11,087.1 ± 3,147 | 2,323.6 ± 738.2 | 4,725.7 ± 1,352.6 | 6,651.5 ± 1,712.8 |  |  |  |  |  |  |
| ***CKX9*** | 9.6 ± 3.2 | 3.1 ± 1.2 | 28.1 ± 4.2 | 35.9 ± 17.2 | 62.7 ± 12.3 | ***PHT::ZmCKX1*** | 255.1 ± 8.9 | 883.7± 181.1 | 112.4 ± 25.9 | 218.0 ± 87.8 | 330.0 ± 36.5 |
| ***CKX11*** | 11,282.5 ± 5,413.0 | 5,368.1 ± 1,990.4 | 24,513.7 ± 2,702.3 | 7,628.1 ± 2,117.4 | 22,482.4 ± 2,702.3 | ***PHT*** | 2.7 ± 1.7 | 0.5 ± 0.2 | 0.4 ± 0.2 | 4.1 ± 1.5 | 4.2 ± 3.7 |
| ***PHT::ZmCKX1-2 (T0 generation)*** | | | | | | | | | | | |
| ***CKX1*** | < 1 | 2.5 ± 0.1 | < 1 | 1.5 ± 0.8 | < 1 | ***IPT1*** | 146.7± 47.4 | 164.2 ± 22.4 | 152.9 ± 21.9 | 95.3 ± 17.6 | 115.9 ± 4.6 |
| ***CKX2.2*** | 6,168.0 ± 1,113.5 | 1,473.2 ± 505.4 | 1,846.2 ± 784.0 | 1,819.5 ± 410.3 | 463.4 ± 189.0 | ***IPT3*** | 12.1 ± 7.6 | 61.7 ± 25.3 | 103.1 ± 24.6 | 60.9 ± 16.3 | 127.9 ± 18.5 |
| ***CKX4*** | 412.2 ± 196.1 | 110.7 ± 15.3 | 1,039.4 ± 423.8 | 439.6 ± 65.7 | 842.3 ± 152.5 | ***IPT5*** | 1,378.5± 45.0 | 466.7 ± 49.6 | 729.3 ± 25.7 | 682.0 ± 84.7 | 1558.0 ± 334.7 |
| ***CKX5*** | 3,203.2 ± 402.1 | 426.4 ± 188.0 | 128.3 ± 27.9 | 518.5 ± 255.9 | 765.6 ± 336.1 | ***IPT7*** | 13.9 ± 3.4 | 37.0 ± 12.1 | 62.6 ± 10.7 | 73.0 ± 9.3 | 9.8 ± 2.5 |
| ***CKX7*** | 24.9 ± 11.9 | 11.4 ± 4.1 | 48.9 ± 5.0 | 56.0 ± 5.6 | 106.1 ± 20.8 | ***IPT10*** | 120.2 ± 36.5 | 237.0 ± 86.0 | 66.9 ± 10.7 | 33.7 ± 10.8 | 55.0 ± 9.1 |
| ***CKX8*** | 5,408.3 ± 1,219.1 | 15,715.7 ± 4,190.0 | 6,892.8 ± 460.8 | 5,694.5 ± 1,872.6 | 4,647.5 ± 838.9 |  |  |  |  |  |  |
| ***CKX9*** | 36.6 ± 23.0 | 4.5 ± 2.3 | 21.9 ± 4.7 | 27.8 ± 14.3 | 62.3 ± 12.2 | ***PHT::ZmCKX1*** | 127.5 ± 17.8 | 2,796.4 ± 383.3 | 217.1 ± 50.5 | 109.5 ± 11.2 | 143.3 ± 51.9 |
| ***CKX11*** | 42,670.9 ± 3,051.0 | 5,061.3 ± 1,226.9 | 6,317.8 ± 1,051.8 | 1,993.1 ± 533.0 | 1,927.9 ± 567.1 | ***PHT*** | < 1 | 1.6 ± 1.2 | < 1 | 1 ± 0.8 | 1.3 ± 1.1 |
| ***PHT::ZmCKX1-3 (T0 generation)*** | | | | | | | | | | | |
| ***CKX1*** | < 1 | < 1 | < 1 | < 1 | < 1 | ***IPT1*** | 19.4 ± 2.6 | 106.7 ± 13.8 | 93.2 ± 30.2 | 81.2 ± 8.3 | 13.5 ± 4.8 |
| ***CKX2.2*** | 27.8 ± 6.3 | 57.9 ± 2.7 | 46.1 ± 13.7 | 6.0 ± 1.3 | 23.2 ± 19.2 | ***IPT3*** | 14.1 ± 10.5 | 13.8 ± 0.9 | 87.2 ± 8.9 | 21.6 ± 2.8 | 190.3 ± 43.3 |
| ***CKX4*** | 70.8 ± 43.6 | 185.2 ± 23.7 | 616.4 ± 121.7 | 504.6 ± 36.5 | 1,083.4 ± 251.2 | ***IPT5*** | 729.0 ± 495.7 | 651.5 ± 190.9 | 949.8 ± 240.1 | 1,334.0 ± 421.9 | 5,567.4 ± 332.5 |
| ***CKX5*** | 337.3 ± 64.3 | 72.7 ± 25.2 | 11.5 ± 3.1 | 16.3 ± 6.4 | 35.8 ± 25.8 | ***IPT7*** | 1.5 ± 0.1 | 21.2 ± 7.4 | 14.4 ± 2.8 | 4.0 ± 1.1 | 9.1 ± 1.1 |
| ***CKX7*** | 10.8 ± 4.5 | 46.1 ± 5.3 | 126.9 ± 7.7 | 59.5 ± 0.4 | 163.7 ± 47.3 | ***IPT10*** | 33.2 ± 7.0 | 65.3 ± 4.3 | 41.9 ± 2.2 | 7.7 ± 3.4 | 8.4 ± 1.6 |
| ***CKX8*** | 892.3 ± 359.6 | 3,704.8 ± 1,185.2 | 2,879.3 ± 1,046.5 | 2,363.7 ± 1,515.6 | 8,622.3 ± 4,389.2 |  |  |  |  |  |  |
| ***CKX9*** | < 1 | 1.5 ± 0.4 | 2.8 ± 0.6 | 13.0 ± 10.2 | 64.7 ± 12.2 | ***PHT::ZmCKX1*** | 14,710 ± 90 | 23,844 ± 1,807 | 5,913 ± 914 | 5,992 ± 814 | 1,370 ± 334 |
| ***CKX11*** | 3,192.3 ± 2,067.6 | 9,441.4 ± 6,922.4 | 8,323.5 ± 5,233.8 | 2,154.4 ± 230.8 | 7,987.1 ± 543.5 | ***PHT*** | < 1 | < 1 | < 1 | < 1 | < 1 |
| ***CTRL*** | | | | | | | | | | | |
| ***CKX1*** | < 1 | 2.1 ± 0.6 | < 1 | 1.0 ± 0.4 | 1.4 ± 0.7 | ***IPT1*** | 67.4 ± 13.2 | 82.6 ± 13.5 | 164.4 ± 42.1 | 65.4 ± 5.2 | 153.0 ± 34.3 |
| ***CKX2.2*** | 9,597.2 ± 2,291.8 | 275.3 ± 53.1 | 373.2 ± 73.3 | 1,637.0 ± 163.1 | 285.4 ± 99.7 | ***IPT3*** | 5.7 ± 1.8 | 16.7 ± 4.7 | 44.9 ± 13.3 | 15.2 ± 5.7 | 69.0 ± 20.3 |
| ***CKX4*** | 149.9 ± 99.2 | 93.3 ± 12.8 | 310.2 ± 76.8 | 302.6 ± 44.6 | 446.8 ± 122.2 | ***IPT5*** | 439.0 ± 116.8 | 477.3 ± 146.6 | 518.0 ± 85.8 | 1,538.9 ± 386.6 | 955.2 ± 261.1 |
| ***CKX5*** | 3,235.8 ± 856.6 | 403.8 ± 102.5 | 111.4 ± 26.3 | 3,473.4 ± 1085.2 | 189.4 ± 62.3 | ***IPT7*** | 26.2 ± 6.5 | 8.5 ± 3.2 | 53.0 ± 20.4 | 14.5 ± 5.5 | 21.9 ± 5.2 |
| ***CKX7*** | 19.3 ± 6.4 | 11.5 ± 5.3 | 36.1 ± 12.6 | 44.4 ± 13.0 | 92.1 ± 2.5 | ***IPT10*** | 36.4 ± 14.1 | 116.2 ± 34.2 | 69.6 ± 30.5 | 53.4 ± 12.5 | 63.0 ± 22.1 |
| ***CKX8*** | 4,428.1 ± 1,413.1 | 13,018.4 ± 4,110.5 | 6,203.1 ± 1,967.3 | 2,354.0 ± 462.6 | 11,534.9 ± 1,642.3 |  |  |  |  |  |  |
| ***CKX9*** | 8.8 ± 3.6 | 11.9 ± 6.2 | 10.2 ± 2.7 | 48.8 ± 13.3 | 529.6 ± 171.6 | ***PHT::ZmCKX1*** | < 1 | < 1 | < 1 | < 1 | < 1 |
| ***CKX11*** | 16,797.6 ± 6,425.9 | 30,676.4 ± 3,306.4 | 20,134.0 ± 5,925.2 | 5,122.6 ± 2,619.9 | 7,944.7 ± 937.1 | ***PHT*** | < 1 | < 1 | < 1 | 2.3 ± 1.2 | 6.1 ± 3.5 |

Abundance is expressed as number of transcripts per ng of total RNA ampliﬁed by qPCR with respect to primer pair efﬁciency. RNA from two biological replicates was transcribed in two independent reactions, and PCR was performed in duplicate. Mean values ± standard deviations are shown.
