# Supplementary material for: Overexpression of Cytokinin Dehydrogenase Genes in Barley (Hordeum vulgare cv. Golden Promise) Fundamentally Affects Morphology and Fertility
Source: PLoS One. 2013 Nov 15;8(11):e79029. doi: 10.1371/journal.pone.0079029 (PMC3829838; doi:10.1371/journal.pone.0079029)
Supplement: Text S1 — DNA genomic sequences of predicted HvIPT genes generated from a rough draft of the barley genome. Shaded sequences indicate start and stop codons, while intron sequences are underlined. (DOCX) [file pone.0079029.s011.docx]

**>HvIPT1**

GGCGAACACCCGTCGTCTCTGTAGAGAAAAAGACCCCTCTAAAATCTGCCCTCGAGATGCCCCACCTCGCCGCACCTGCCTCTCCGCCGCCGGCTCCAAACCTAATGTCTGACGCCCGCGAGGCTGCCTCCTCCTCGCCGCCGTCGGAAAGGGGGCGGAGGAAGGCAGTGGTGGTGGTCATGGGCGCGACGGGCGCCGGCAAGTCCCGGCTTGCCGTTGACCTGGCGGGCCACTTTACCGGCGTCGAGGTGGTCAGCGCCGACTCCATGCAGGTCTACCGCGGTCTCGATGTCCTCACTAACAAGGTCTCCCTCCATGAGCAGAACGGTCTCTCCTCTCGAACACTCCACAGTCCCGCCTTATATATATTTTTCTCTTCAAAAATTGCAAATTCAGTTCCGTTCAGTTTGAGCTGAGCGACGCTATATTTGGCGTGTCTGCAGGTGTTCCTCACCATCTCCTTAGCATCGTCGATCCTTCCGTGGAGTTCACTTGCCGCGATTTCCGTGATCATGCCTTGCCTGTAAGCGTACATCCGGTGTTACTGATGCAAGCTCGTTTCAGTCTTGTACCAGACTTCCAACTGAGTTACCGTACCATGTTCATTCTAAATGGCCGCAGATTATAGAAGATATATTGGATCATGGCGGCCTGCCTGTTATTGTTGGCGGCACAAACTTCTATATACAGGTCCATAGTTCAGCACGTGTCAATTTCCATATCAGGAGTTATTCACACATGTTCATAGTTCAATTCTGGTTGATATCCTTATCTTAAGTTAATTGTGTGTCTGATTTACATTTTTGTGGGCTCTTATGATGGCCTTAGGCTCTTGTTAGTCCATTCCTTTTTGAGGATATGTCAGAAGATATGCAGGGTTGCACTTTGAGTGACCAACTTGATGATATAGGTGAATATTGAAACCATAACACTTGCTCCTTATTCATCGTGTGCCTGTTCTGTATTCATGTTTCATTTATTTGCCTATCATATCTTCAATCTGCTACTCAGGCCTTGCCACGGATGATGTTAGAAGTGGGTATGAACACTTGAAGGAGATTGATCCTATTGCAGCACAAAGGATCCACCCAAACAACCATCGAAAAGTAAGGGTGTTGCACATTCCTACCATCTCCTGGTCACTCAGCCAGTCGTTCTTCATCTGTAGCATGCCCTCACTTTCCTCATGCCATTCCCCTATTGAACAGATAAACCGTTATCTTGAGTTATATGCAACCACAGGTGCACTACCAAGCGATCTTTTCCAAGGAGAAGCTGCAGAAGTGAGTAATTTTTGTAATTTTTTTAATGAATCATTCAGTATATATGCCATGAATATGAACTACTAACTGCTACAACACATTTTTTGCGCATGAGGTTAGACTTTCCTGTTTCAATATGGATGACCTAAACAAGATTGTTTGTTGTCAGGACAAGTGGGGCCGGCCTACTAGTTCCAGATTTGACTGTTGTTTCCTGTGGGTAGATGCCGAGCTTCATGTTCTGGATAATTATGTCAATGAAAGAGTCGATTGCATGATTAATGCTGGCCTGCTTGATGAAGTGCGCAATATATATAATCCAGGTGCCGTTTATACCCAAGGTCTGCGGCAGGCCATTGGGGTTCGTGAATTTGATGAGTTTTTCATATCATATTTTACAAAGAAAGAACCTAATAATGAGATGAAAGCTGGTATGCTTGATCTCCATGATGATAAGCTGAAAAACTTGTTGGATGAAGCTGTCTCCCAGCTAAAAGCAAACACACGAAGACTTGTTCGACGCCAAGTAAACACTCCACTTCCTCTGCATCTTTATTCTTACATTTTTTTAGAAGGTCTGAGAAAATAATTAGTACTAGACTGCAAATCTTTATCGCAGAGACGGAGGCTACACCGGCTGAATAAAGACTTCGGGTGGCATTTGCATCACATCGATGCAACAGAAGCATTCCAATGTAAGTCCATTTGTATGTATTAGCCCATAGTGTTGAATTGGCCTGCTTCGATGTGTTCTTTTCCTTCACATGTAATGTATATTTTGCTAGGTACCACTGGTGACTCGTGGCACGTCAAGGTTATAAAACCTTGCGTAGATACTGTCAGGAGATTCTTGTCTAATGATACAAGTTTGGCAAGTAAAGATTGTTCAAATGATGGTGGAGGGACTAGGTTGGCCTCAAGAGAGTTATGGACTCAATATGTTTGTGAGGTGATGTTTTTGTTTTATGTTCTTAAGAGGTGGTTGTGGTTATGCTTTGGTTGTCCTATGATGATTGCTTGAAGTATCTGATGCTTGCCTTTTATCATTTCCCTCGATGTATTCGTTAATAAAAACATGGATCTGACAAACTTCTGCCCTTTCTGAATCGTGTGTTATAGGCCTGCGACAACCGGATCCTTCGAGGGGCACACGAGTGGGAACAACACAAGCAAGGGCGAGGCCACCGGAAAAGAACGCAGCGCTTGAAGCGGAAGAGTAAGATGAAGAGTACATCGTCTGAAGCAGAAGTATAACTGTATGAGCATCAGTTTTAGTTTCGGTGTAGATGGAACACCATTTAGATTGGTTTTGAGTCTTATGGAGGAGACTGTGGAAGGGGATTGATAAGGGCACGGATCCAAACACTAGTTCTGATCCTTAAAGGCGTGTTTGTATGCTGCCTGACTTCATATTGCCGCAAACAACAGTTCATCTTAATTTGTGCGCATTCTGAACATGCCATCTAATTGTTCATAGAGCTGGTGAATATCGTCAAGGTGAATTTAAAAGAAATCAACAGTGGTTCGAAAACTAAATAAATGAAAGAGATAAAAGGTAAATCCATGAAACATTAGAATGCAGATCATAATATTTTAGAAAGGAAGATCCAGGACGACGGTCCAAACAGTTTTAT

**>HvIPT2**

ATAAAAGCCGGCGTCCCGGTCGCGTCACTGTCAACAATCCGGCAAACACGCCCTCCTCTTACGTACGAACAATCCATGGGCGCCGCCGTGATGGAGCAGCGACGCGGCGGCAAGCCCAAGGTGGTCTTCGTGCTGGGCGCCACGGCCACGGGCAAGTCCAAGCTGGCCATCTCCCTGGCGGCGCGCTTCGGCGGCGAGGTGATCAACTCGGACAAGATCCAGGTGTACGCTGGCCTCCCCGTGATTACCAACAAGGTGACGGACGAGGAGTGCGCGGGCGTGCCGCACCACCTCCTCGGCTCTGTGCCGTGCCCCGACGCTGACTTCACCGTCGACGACTTCTGCCGGGAGGCGGCTGACGCGATCAAGCGCGTTCTCTCCCACGGCGGCCTCCCGGTGGTGGCCGGCGGCTCCAACAGGTACGTCGAGGCGCTTGTCGACGGAGATGGCGGCGCCTTCCGCTCCTCACATGACTGCCTCTTCGTCTGGCTTGACGCCGCGCCAGAAGTCCTCCGCCGGTCCACAGCGGTACGGGTGGACGACATGGTGCGGCGCGGACTGGTGGAAGAGGCTCGCGCTGCGTTCGACCCGGATGCCAGCTACACCAGGGGCGTGCGTCGCGCCATCGGCCTGCCGGAGATGGACGCGTACCTGCGGCGTGCGAGCGACGGCGATGACGAGGCGATGCTGGGGAGAGCGGTGGAGGAGATCAAGGTTAACACGTTCGGGCTGGTGCTGGAGCAGGTGGAGAAGATCCGGCGGCTGAGCACGCTGGAGGGATGGGACGTCCGGCGGGTGGACTGCACGGAGGTGCTGGCGCGGATGGTGGACGGCGAAGGGGTGCAGGAGCTATGGAGGAAGGTGGTCTGGGAGCCCGTCGAGGACATGGTGCGGACCTTCGTCATCGCGGAGAAGAGCTGTGGGAAGATCAGCCTGCTGGCCTAGAGTCTAGAGCTATCTTATATTTTGGCGCCCTGTGTTAGGTTTGAATTGTCCGCCTGGTTTCGTAGTAAACGTGTCGAAGAAATGTGAGGAGCTTCCTACATATATACTCCTTCATTGCCGTTTCCAATTATTCTGTCCAGAATTACCTATTTCGATCTTTCACTACATGAAAATTACTTGTGGCCGTGC

**>HvIPT3**

TTGCTTTAGTATTTAAATCGAGTGTCCTTGCGGCTTGCGCCCAAGTCGCTCCCTATATAAGCCAGTCCCCGGTGCTAGAGTGACTGGCGGGGGGCACGGGATTGAGCCGCCTATATGAACAGCCTCCTCAGCAGGATCGGCGGCGCCGTCCGCTCACCGATGGCGTCCGCGGCCATCGCTGGCGTCGGCGGCACACATAGGCGGCCGCCGTGGCTCAGTGTGGAAATGGAAAGGACAAGAGCCAGGGGTTGTTGCGGCTGCAGCTGTAGCGGCACCAAGATGGAGACGGAACGTGGGGACACGAGGCTCGTGGTGATCGTGGGGGCAACAGGCACGGGCAAGACCAAGCTGTCCATCGACGCGGCACAGGAGCTCGATGGCGAGGTGATGAACGCCGACAAGATCCAGCTTTACCACGGCCTCGACGTCACCACCAACAAGGTGATGCCCGCCGACCGCCGCGGCGTCGCCCACCACCTCCTCGGCGCCGTCCCCGCCGACGCCGGCGAGCTGCCCGCCTCGTCCTTCCGCTCGCTCGCCGCCGCCAAGGCGGCCGACATAGCGGCGCGCGGGCGCGTGCCAGTGGTGGCCGGCGGCTCCAACTCTCTCATCCACGCCTTCCTCGCCCAGCGATTTGACGGCCACGCCCCTCGGGACCCTTTCGCCGCTACGGCGACCAGATACCGGCCGGCGCTCAGGTTCCCGTGCTGTCTCCTCTGGGTCCATGTCGACGAGGCAGTTCTCGACGAATACCTCGATCGGCGCGTCGACGACATGCTGGGTCAGGGCATGGTGGAGGAGCTCCGCGAATACTTCGCGACAACGTCGGACTCCGAGCGGGCCTCGCACGCCGGGCTGCGCAAAGCCATCGGCGTGCCGGAGATCGGAGACTACCTCGCTGGGCGTAAGAGCCTCCGCACGGCGGTAGACGAGATCAAGGAGAACACGAGGGTGCTCGCGGCGGCGCAGGTCGGCAAGATCCGGCGCATGGCAGACGGCTGGGGCTGGCCCGTTCGGCGCCTCGACGCCACCGGCACCGTCCTCGCGCGGCTCGCGGGCGCCGGGCGCGACGCCGAGGCCGCGGCGTGGCATCGCGACGTGCGCGGGCCGGGCCTCGCCGCCATGCGGCAGTTCCTGCGCAGTCAAGGACTGCCCGGACACAACGACGACGCCGATGACGAGGACGGCTTGAGACAATGCCGTGGCATGGTGGGGTAACAGGAACATGAAAAAGCCTGAGAGCTTTTCGGGTTCGGGCGCCACGACGAGGCCGCGGCCGCGGCGTGGGATCGCGACATGCCCGCCCGGGCCGGGCGTCGCCGCCATGCGGCAGTTCCAGCGCAGCCAA

**>HvIPT4**

TGTCACTTTATTGAGACTTAGACTGAGATCTATGCACACCCTAGTATTTTTTCTTCTCTGAGTGAGTAGCAGTAAACATGGGTTACCCTCGTATTGTTCCTTGAGCAGCTGCTGATCAATGGAGTGCAAGGGCGTGAACGGCCAGTCCCCCAAGCCCAAGGTTGTTTTCGTGCTTGGAGCCACGGCCACCGGAAAGTCCAAGCTCGCCATCTCCCTCGCCCGGCGCTTCGGCGGCGAGGTCATCAACTCCGACAAGATCCAGGTGTACGACGGCGTGCCAATACTGACCAACAAGGTGACCGAGGAGGAGAGCGCCGGCGTGCCGCACCACCTCCTGGGTGGCGTGCATCCCGACGCCGACTTCACCGCGGACGACTTCCGCCGCGTGGCCGAGGCCGCCATCTCCCGCGTCATCTCCGCGGGCCGCCTCCCCGTCGTTGCCGGTGGGTCCAACAACTACGTAGAGGCGCTGGTGGAGGCCGACGGCGCCGCGTTCCGCGCCGCCCACGACTGCCTCTTCCTCTGGCTCGACGCCGCGCCGGGTTTGATGGAGTGGTACACCGGCCTGCGCGTCGACGACATGGTGCGGCGCGGCCTGGTGGACGAGACGCGCGCCGCCTTCGAGGAGGGCGCGGACTACACCCGCGGCGTGCGGCGGGCCATCGGCCTCCCTGAGATGCACGAGTACCTGCGGGCGGAGCGGGAGGGCGCCGTCGGCGCGGCCGAGATGGCGGCCCTGCTCGAGCGCGCCGTGCGCGAGATCAAGGCCAACACGTTCGGCCTCGTCCTCCAGCAGGCGGCCAAGATCCGCCGCCTCAGCACGCTGGAAGGCTGGGACGTCCGCCGCGTCGACGCCACCGCCGTCTTCGCGTCCATGGCCGAGGGCTCGGGCGGCCACAAGGAGGTCTGGGAGTCGGCCGTGTGGGCACCGTGCCAAGAGATGGTGCGCCTTTTCCTCCATGCTGAAGCTGCCACTCTGCCAACTGATCTCGAGGTGGATGAGGCTATGGCTGGGCTTTCCCTCGGTGTCTCGGTTATCCCCGTTGCTGCAGTCGGTGACGTCGGCGACGATGATGTAAGCGTTGTCCTGACCCCGCCAACAGTACTGCACGAGCATGAGAATGAAGGATCCATAAACAAGGATGGTGCTGCCGGCGTTGTACTCAATGGCACCCACGACGTCGTGGACAAGGATCCTGCCTGCGGTGGCGGCGATGGTGAGGATCATGGAAGCAATGCCGGCGTTGCTCAGGCTGCTCCGGCAGCAGCTGGAGCTAAGCCGGACGACACAGCCTAAGCTAGCTGGGAACATGCATGCATGCATGCATGGCTTCATTACCGGCTTTGTCGCATTCCATGGTGCTTGGAGTTCTCCACTCGTGCTTTGGACGTACTCCTCCACAGTCGGTTGCATGCAAGCATGGGAGATAGTGGCACAAGTCTCATGAGCTTAAGCTAGCTTTACTGTTTAATTTATCTCTCGTTTTCT

**>HvIPT5**

CAGAGCAGCATTCCAAAACCAGAGGCCACCGCACTCTCTAAGCGTGCGAGCGGCGAGCGATCGAGCCTAGCGCGTGAACCGAGCATGCCGATGATCTCCGTGGCGCCGCCGCCGGCCCTGGCCTTCCCGAGGCTGACGCTGACGATGCCGCCGCCGTTGATCGCGCTCCCGGACCGCGCCGACGCGCCGCGCCCGCCCCAGCCCCTCGTGGTCCGCCACGCCGCCGCCAAGCACAAGGCCGTCGTCGTCATGGGCGCCACGGGCACCGGCAAGTCCCGCCTCGCCATCGACCTCGCGCTGCGCTTCGGCGGCGAGGTCATCAACTCGGACAAGATGCAGGTCTACACCGGCCTCGACGTGGCCACGAACAAGGTGTCCCCCCGCGAGTGCGCCGGCGTGCCGCACCACCTGCTTGGCCTCGTCGACGACCCCGACCAGGACTTCTCCGCCGCCGACTTCCGCGGCGAGGCCACGCTCGCCGCGGGCTCCGCCTCGGCGCGCGGGTATGTCCCCGTTGTTGCCGGCGGCTCTAACTCGTACGTTGAGGAGCTCGTGGAGGGCGACCGCCGCGCCTTCCGGGAGCGCTACGACTGCTGCTTCCTGTGGGTGGACGTGCAGCTCCCCGTGCTGCGCGACTTCGTGGCCCGCCGGGTCGACGACATGTGCCGCCGCGGCCTCGTCGACGAGGTGGCCGCCGCCTTCGACCCGCGCCGCACAGACTACTCCCGCGGCGTCTGGCGCGCCATCGGCGTCCCAGAGCTTGACGCCTATCTTCGCTCCACCGGCGTCGGCGAGGACGAGCGCGCGCGCATGCTCGCCGCCGCCGTCGACGAGATCAAGGCGAACACGTCGCGCCTCGCGTGCCGCCAGCGCGGCAAGATCCAGCGGCTGGCGCGCATGTGGCGCGTCCGCCGCGTCGACGCCAGCGAGGTGTTCCTGAAGAGCGGCGCCGCCGCCGACGAGGCGTGGCAGCGGCACGTCGCCGCGCCCTGCATCGACGCCGTCCGGTCGTTCCTGCTCGAGGACCAAGAATGCAGCATGGTCGCCGCCGCCGGCAATGCCTCCGTCTTCGCCTCCGCCGCCGGCAATGCATCCGTCTTCGCCTCCACCGCCGTCGCCTAGCTAATCCAAACACACTCCCTACATACGTACTTACCAAAACACAAGAAACAACCTGCACGAACGAACGCTTCCCGAACTAGCTTCGGCGCCAGCTCCACAGCCCAACCGCCATTGACGGTCGACCCGGAGCTCGACCGCGAGCTGAGCAAAG

**>HvIPT7**

ATAAATACGAGCCACTTCCGCTAGTTTCATGCACCGCTCGATCTCCCCGAAAATCGTACAGTAACTTCTTCTCAAGCGTGCGCGCGGCAAAACGAACAGACAGACTTCCCTACTCCTTACTCGTGCGTGCATGGCGATGGCGGGCATTAGCACGGCAGCGACCGGCAATGGCAAAGCCAAGGTGGTTATTGTGATGGGCGCCACGGCCACCGGTAAGTCGAAGCTGGCCGTCGACATCGCGCTGCGGTTCAACGGCGAGGTCATCAACTCGGACAAGATCCAGGTCCACGACGGCCTCCCCGTGGTCACCAACAAGGCCACCGCCCGCGAGCAAGCGGGGGTGCCGCACCACATGATCGGTGGGGTACAGCCCGATGCTGACTACACTGCCGCGGACTTCCGGCGCGACGCCACGCGCGCCGTGGAGTCGGTCCTCGCGAGGGGCCGCCTGCCGATCATCGCCGGCGGGTCCAACAACTACCTCGAGGCGTTGCTGGACGGCGAGCTGGGATTCCGCCGGCGGTACGACTTTTGCTTCCTATGGGTGGACGCGGAGACGTCGGTGCTGGAACGCTACGTTGGCGACCGCGTCGACTGCATGGTGGAGCAAGGGCTCGTCGGCGAGGTGCGGGAGTTTTTCCGGGCGGACGCGGACTACTCCCGTGGGATCCGGAGGGCCATAGGCGTGCCGGAGATGGACGCCTACTTCCGGATGGAGACCGCGGGGGCGCTGGACGGAGACGAGGAGCGACGGGCCGGGCTCCTCGAGGCCGCCGTCGACGAGATCAAGGAGAACACATGCAGGCTGGCGTGCAGCCAGCTGCGGAAGATCCACCGGCTGCAATGCCTGCCTGGGTGGAGCATCCGCCGCCTTGATGTCACCAGGGTGCTTTCGCTCAAGGTCGGGAAGAAGAAGGACGAAGAAGCGGAGCGCACCATGTGGGAGGCGGATGTCGTCGGGCCTGCGGCGCGCGTCGTTGAGATGTTTCTTCACCCTCTTGGAGGGATGGTGGCCGAGGTGAGTAGGGATGGGAAGGAGCAAAGCACGGGGGCGAAGCATGCAGCCGTCGTCGCCGGAATCGTCGAAGCGGCCGAGAGGTGTGGTCTGCAGCTACTCGAGACCGGGCCGTCTCGTGGGATTCATCCAAGGAAAGCGGCTGCAGCAGTTTGAGTTTGGAGCTTCTCATCAAATTAACCATACCACGTATTTAGTAGTACTAGCCTGTGAACTCTAATCAATTGTTTTATTCTTTTAATCTAGTTGTGTTCTTCTATCCATTAAGTTATATAAAGGGAAAATCTGACGCATTTTGTCTATACCGTGGGGCGGCTTGCCGACTGATTTGGT

**>HvIPT10**

CGCTCACAACTCAGAACGAACCCCTTATCTTTGTTTTCGCTTCAGTCAATCAATCTCTGCACATGCGCTTCCTCTTCGTTCTGTGATCTCAATCTGAATCAGCTTCTGCAGGTGCCCGCTCTATCATGTGCTGTGAAATGAGGCCGGGGTTTGGGTTTGGGTTCGGGTCCGCGCGGCGCGGCATCTGGAGGACCTGGCCCGCCCTCTGCTCACGGAACCAGCAGCGCTTCGCGTCGTCTCTTGACGTCATGGCTGCCACAATGCCGGCACCAAACCAAGCAAGGAAGAGGAGCAAGGTCATCGTGATATCAGGACCCACCGGCGCTGGGAAGAGCAGGCTCGCCTTGGAGGTGGCCAGGAGGCTCGGGGGAGAGATCATCAGTGCCGACTCAGTCCAGGTGTACCGCAGCCTCGACGTCGGTTCCGCCAAGCCGTCTGCTTCGGAGATGAGCATGGTGCCGCACCACCTGATCGACATCATGCACGCGTGCGAGGACTACTCGGCTGGGATGTTCTTCAGTGACGCGCGAAGGGCGACTCAAGATGTTCTTGGCAGGGGCGCTGTGCCAGTTGTCGCAGGAGGGACTGGGCTGTACCTGCGGTGGTTTATCTACGGCAAGCCAAATGTTCCACAATCGTCCTCCGACATCATATCGAGTGTGTGGTCTGAGCTTGCTGGCTTTCGTGAGAGCGGTCGGTGGGAAGAAGCGGTGGAGCTCCTGCTCAAGGCCGGGGACTCTGAAGCTCGGGACTTAGATACGAATAACTGGGCCAGGTTAAGCAGAAGGCTCGAGATCATCAGGTCATCAGGTTCCCCTGCATCTGCCTTCACCTTACCATACAGCTCATTCCAGAAGCAGCAAGACACCAAGCTAACTGATTCCCCAAGCGATGATGCAACCTGTGAAACGAAGGAACTGGATTATGACTTCCTATGTTTTTTCCTCGCATGTCCACGGGTTGAACTCTACAGATCAATTGATTTGAGGTGTGAGGAAATGCTGGTTGACACAGGAGGCCTTCTTTCAGAAGCCTCATGGCTTCTTGATATCGGGCTGCAGCCAAATATGAACTCCGCGACCCGTGCTATTGGTTACAGGCAAACCATGGAGTACCTGCTGCATTGCAGACAAAACGGAGGCAGTAGCTCCCCAGAGGAGTTCTTGGAGTTCCTAACCAAGTTTCAGCAGACTTCTAGGAACTTCTCAAGGAGGCAAATGACCTGGTTCCGCAACGAGAAGATTTACCAGTGGGTTGACGCGTCACAGCCTTTTGAAGAAATAGTGCAATTCATCTGTGATGCTTACAATGGCTCTGATGCATTGGTGCTGCCTGAATCTCTTGAAATGAAAAGGGAAAGCTGCCTGCACACGAGCAAGGATCTCAAAACCTATCGTTCAGAGAACAGGGTGTTCCTTGGGCATGAAGACTGTCGACACATTTTGGATTGGATTAGGAGGACGCAGGGGAAGTGAGGTCTAGTTCTTGTCACTTCTCATACTTTGTTTTGTTTCCACCAGAAAATTTATCCGGCTATAGAATTTTGCTTTTTGAGAGATTAC
